# INOSITOL (1,3,4) TRIPHOSPHATE 5/6 KINASE1-dependent inositol polyphosphates regulate auxin responses in Arabidopsis

**DOI:** 10.1093/plphys/kiac425

**Published:** 2022-09-19

**Authors:** Nargis Parvin Laha, Ricardo F H Giehl, Esther Riemer, Danye Qiu, Naga Jyothi Pullagurla, Robin Schneider, Yashika Walia Dhir, Ranjana Yadav, Yeshambel Emewodih Mihiret, Philipp Gaugler, Verena Gaugler, Haibin Mao, Ning Zheng, Nicolaus von Wirén, Adolfo Saiardi, Saikat Bhattacharjee, Henning J Jessen, Debabrata Laha, Gabriel Schaaf

**Affiliations:** Department of Plant Nutrition, Institute of Crop Science and Resource Conservation, Rheinische Friedrich-Wilhelms-Universität Bonn, Bonn 53115, Germany; Department of Physiology & Cell Biology, Leibniz-Institute of Plant Genetics and Crop Plant Research, Gatersleben 06466, Germany; Department of Plant Nutrition, Institute of Crop Science and Resource Conservation, Rheinische Friedrich-Wilhelms-Universität Bonn, Bonn 53115, Germany; Department of Chemistry and Pharmacy & CIBSS–The Center for Biological Signalling Studies, University of Freiburg, Freiburg 79104, Germany; Department of Biochemistry, Indian Institute of Science, Bengaluru 560012, Karnataka, India; Department of Plant Nutrition, Institute of Crop Science and Resource Conservation, Rheinische Friedrich-Wilhelms-Universität Bonn, Bonn 53115, Germany; Laboratory of Signal Transduction and Plant Resistance, Regional Centre for Biotechnology, NCR-Biotech Science Cluster, Faridabad 121001, Haryana, India; Department of Biochemistry, Indian Institute of Science, Bengaluru 560012, Karnataka, India; Department of Plant Nutrition, Institute of Crop Science and Resource Conservation, Rheinische Friedrich-Wilhelms-Universität Bonn, Bonn 53115, Germany; Department of Plant Nutrition, Institute of Crop Science and Resource Conservation, Rheinische Friedrich-Wilhelms-Universität Bonn, Bonn 53115, Germany; Department of Plant Nutrition, Institute of Crop Science and Resource Conservation, Rheinische Friedrich-Wilhelms-Universität Bonn, Bonn 53115, Germany; Department of Pharmacology, Howard Hughes Medical Institute, University of Washington, Seattle, Washington 98195, USA; Department of Pharmacology, Howard Hughes Medical Institute, University of Washington, Seattle, Washington 98195, USA; Department of Physiology & Cell Biology, Leibniz-Institute of Plant Genetics and Crop Plant Research, Gatersleben 06466, Germany; Medical Research Council Laboratory for Molecular Cell Biology (MRC-LMCB), University College London, London WC1E 6BT, UK; Laboratory of Signal Transduction and Plant Resistance, Regional Centre for Biotechnology, NCR-Biotech Science Cluster, Faridabad 121001, Haryana, India; Department of Chemistry and Pharmacy & CIBSS–The Center for Biological Signalling Studies, University of Freiburg, Freiburg 79104, Germany; Department of Biochemistry, Indian Institute of Science, Bengaluru 560012, Karnataka, India; Department of Plant Nutrition, Institute of Crop Science and Resource Conservation, Rheinische Friedrich-Wilhelms-Universität Bonn, Bonn 53115, Germany

## Abstract

The combinatorial phosphorylation of *myo-inositol* results in the generation of different inositol phosphates (InsPs), of which phytic acid (InsP_6_) is the most abundant species in eukaryotes. InsP_6_ is also an important precursor of the higher phosphorylated inositol pyrophosphates (PP-InsPs), such as InsP_7_ and InsP_8_, which are characterized by a diphosphate moiety and are also ubiquitously found in eukaryotic cells. While PP-InsPs regulate various cellular processes in animals and yeast, their biosynthesis and functions in plants has remained largely elusive because plant genomes do not encode canonical InsP_6_ kinases. Recent work has shown that Arabidopsis (*Arabidopsis thaliana*) INOSITOL (1,3,4) TRIPHOSPHATE 5/6 KINASE1 (ITPK1) and ITPK2 display in vitro InsP_6_ kinase activity and that, in planta, ITPK1 stimulates 5-InsP_7_ and InsP_8_ synthesis and regulates phosphate starvation responses. Here we report a critical role of ITPK1 in auxin-related processes that is independent of the ITPK1-controlled regulation of phosphate starvation responses. Those processes include primary root elongation, root hair development, leaf venation, thermomorphogenic and gravitropic responses, and sensitivity to exogenously applied auxin. We found that the recombinant auxin receptor complex, consisting of the F-Box protein TRANSPORT INHIBITOR RESPONSE1 (TIR1), ARABIDOPSIS SKP1 HOMOLOG 1 (ASK1), and the transcriptional repressor INDOLE-3-ACETIC ACID INDUCIBLE 7 (IAA7), binds to anionic inositol polyphosphates with high affinity. We further identified a physical interaction between ITPK1 and TIR1, suggesting a localized production of 5-InsP_7_, or another ITPK1-dependent InsP/PP-InsP isomer, to activate the auxin receptor complex. Finally, we demonstrate that ITPK1 and ITPK2 function redundantly to control auxin responses, as deduced from the auxin-insensitive phenotypes of *itpk1 itpk2* double mutant plants. Our findings expand the mechanistic understanding of auxin perception and suggest that distinct inositol polyphosphates generated near auxin receptors help to fine-tune auxin sensitivity in plants.

## Teaser

Arabidopsis INOSITOL (1,3,4) TRIPHOSPHATE 5/6 KINASE1-dependent inositol polyphosphates bind to the auxin receptor complex and regulate auxin responses.

## Introduction

The phytohormone auxin orchestrates a plethora of growth and developmental processes, including embryogenesis, root development, and gravitropism (Teale et al., 2006; Lavenus et al., 2013; Salehin et al., 2015; Weijers and Wagner, 2016). Its distribution within plant tissues creates various organized patterns, such as leaf venation (Scarpella et al., 2006), phyllotactic patterning (Reinhardt et al., 2003; Jönsson et al., 2006; Hartmann et al., 2019), and xylem differentiation (Fukuda and Komamine, 1980; Bishopp et al., 2011; Smetana et al., 2019). Auxin perception is mediated by TRANSPORT INHIBITOR RESPONSE1 (TIR1) and AUXIN-SIGNALING F-BOX proteins (AFB1-5), which induce SCF ubiquitin-ligase-catalyzed degradation of AUXIN RESISTANT/INDOLE-3-ACETIC ACID-INDUCIBLE (Aux/IAA) transcriptional repressors to activate AUXIN RESPONSE FACTOR (ARF) transcription factors (Gray et al., 2001; Dharmasiri et al., 2005; Kepinski and Leyser, 2005; Prigge et al., 2020). Surprisingly, in a crystal structure of the auxin receptor complex, consisting of insect-purified ARABIDOPSIS SKP1 HOMOLOG 1 (ASK1)–TIR1 and an IAA7 degron peptide, insect-derived InsP_6_ occupied the core of the leucine-rich-repeat (LRR) domain of TIR1 (Tan et al., 2007). While the functional importance of InsP_6_ in auxin perception remains elusive, this molecule serves as a major phosphate store in seeds and as precursor of InsP_7_ and InsP_8_, inositol pyrophosphates (PP-InsPs) whose *myo*-inositol ring contains at least one energy-rich diphosphate moiety.

PP-InsPs regulate a wide range of important biological functions, such as vesicular trafficking, ribosome biogenesis, immune response, DNA repair, telomere length maintenance, phosphate homeostasis, spermiogenesis, insulin signaling, and cellular energy homeostasis in yeast and mammals (Wilson et al., 2013, 2019; Thota et al., 2015; Wild et al., 2016; Gerasimaite et al., 2017; Shears, 2017). PP-InsPs were also identified in different plant species (Brearley and Hanke, 1996; Flores and Smart, 2000; Dorsch et al., 2003; Desai et al., 2014; Laha et al., 2015). In plants, PP-InsPs have been shown to regulate jasmonate and salicylic acid signaling in Arabidopsis (*Arabidopsis thaliana*; Laha et al., 2015, 2016; Gulabani et al., 2021), target of rapamycin (TOR) signaling in Chlamydomonas (Couso et al., 2016), and to represent potential targets of bacterial type III effector enzymes in pepper (*Capsicum annuum*) and tomato (*Solanum lycopersicum*; Blüher et al., 2017). PP-InsPs were also shown to regulate phosphate signaling in Arabidopsis (Wild et al., 2016; Dong et al., 2019; Zhu et al., 2019; Riemer et al., 2021; Zhou et al., 2021), by promoting the physical interaction between PHOSPHATE STARVATION RESPONSE (PHR) transcription factors with stand-alone SYG1/Pho81/XPR1 (SPX) proteins (Wild et al., 2016; Dong et al., 2019; Ried et al., 2021; Zhou et al., 2021).

Metabolic pathways leading to the production of PP-InsPs are well established in yeast and metazoan. In these organisms, IP6K/Kcs1-type kinases phosphorylate InsP_6_ at the 5-position, generating 5-InsP_7_ (Saiardi et al., 1999; Draskovic et al., 2008), while PPIP5K/Vip1 proteins catalyze the phosphorylation of 5-InsP_7_ to generate 1,5-InsP_8_ (Mulugu et al., 2007; Lin et al., 2009; Zhu et al., 2019). PPIP5K/Vip1 enzymes are ubiquitously found in plants from green algae to monocot and eudicot angiosperms (Desai et al., 2014; Laha et al., 2015). The Arabidopsis PPIP5K isoforms VIH1 and VIH2 were shown to complement *vip1Δ-*associated defects in yeast and to be critical for 1/3-InsP_7_ and InsP_8_ synthesis in planta (Laha et al., 2015; Zhu et al., 2019; Riemer et al., 2021). While canonical IP6K/Kcs1-type kinases are not encoded by genomes of land plants, recent work identified an evolutionarily conserved InsP_6_ kinase activity of Inositol (1,3,4) Triphosphate 5/6 Kinases (ITPKs; Adepoju et al., 2019; Laha et al., 2019; Whitfield et al., 2020), and a role of Arabidopsis ITPK1 in 5-InsP_7_ and InsP_8_ synthesis (Riemer et al., 2021). In agreement with a proposed role of InsP_8_ as a proxy for the cellular phosphate (P_i_) status (Dong et al., 2019; Zhu et al., 2019), *itpk1* mutants display constitutive phosphate starvation-induced gene expression and overaccumulate Pi (Kuo et al., 2018; Riemer et al., 2021).

In this study, we investigated *itpk1*-associated growth phenotypes that appear to be independent of compromised phosphate starvation responses. We find that *itpk1* plants display defects in various auxin-regulated processes and show that highly anionic inositol polyphosphates bind the auxin receptor complex with high affinity. We also provide evidence for a direct interaction of ITPK1 with the auxin co-receptor component TIR1 in planta, suggesting dedicated substrate channeling of ITPK1-dependent InsPs/PP-InsPs to activate auxin signaling. Our study further reveals that ITPK1 and ITPK2 function redundantly to control various auxin-responsive processes in Arabidopsis.

## Results

### ITPK1 deficiency causes a defect in primary root elongation and changes in inositol polyphosphates that are rescued by a genomic *ITPK1* fragment

**Figure 1:**
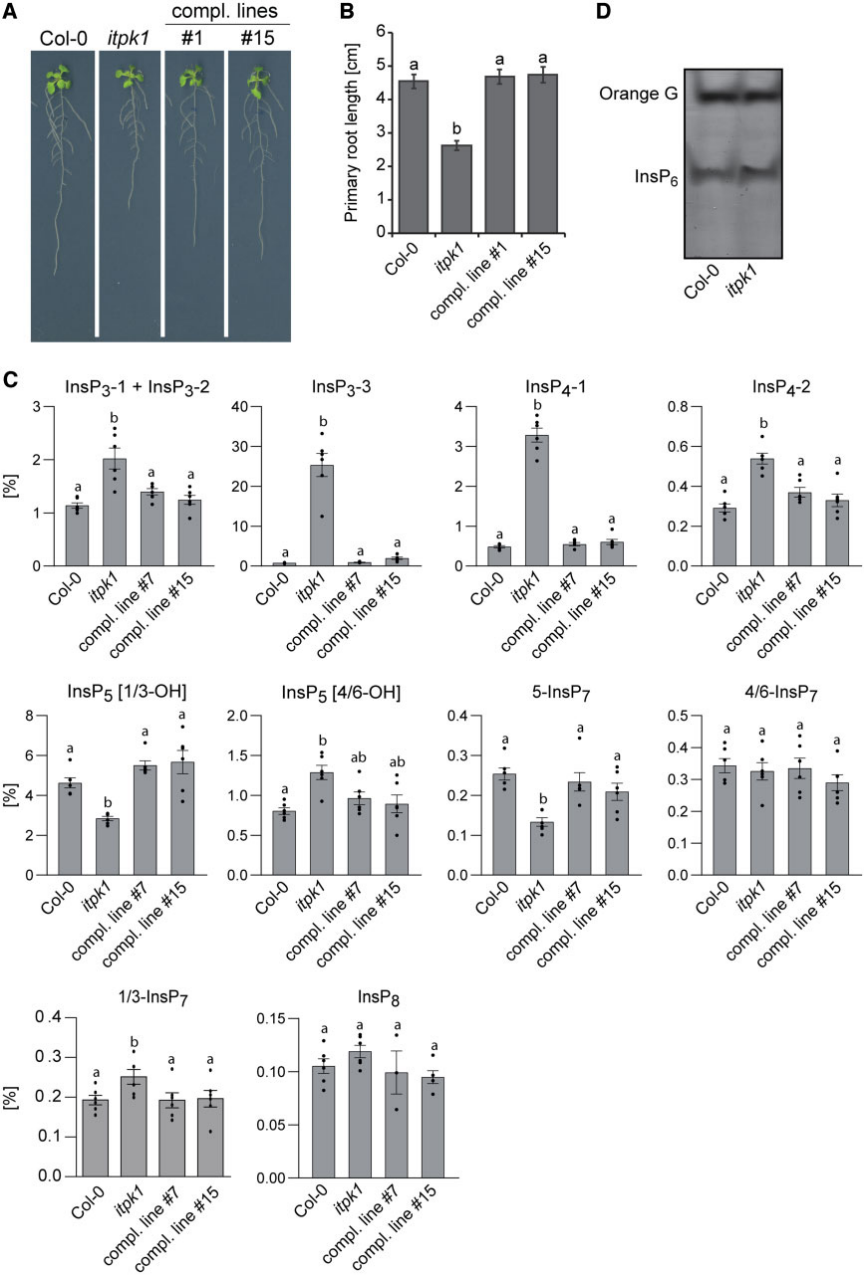
The *itpk1*-associated defects in root architecture and inositol polyphosphate profile are fully rescued by introducing a genomic *ITPK1* construct. A, Primary root elongation of seedlings of wild-type (Col-0), *itpk1* and two independent complemented *itpk1* lines. The latter refer to the *itpk1* T-DNA insertion line transformed with a genomic fragment containing a 1,839-bp region upstream of the *ITPK1* start codon in translational fusion with a C-terminal G3GFP. Seeds were germinated on solidified half-strength MS agar media, supplemented with 1% (w/v) sucrose. After 7 days, seedlings were transferred to fresh media with the same composition and grown for additional 7 days until imaging. B, Complementation of short primary root elongation of *itpk1* plants by an *ITPK1* genomic fragment C-terminally fused to G3GFP. Six-day-old seedlings of designated genotypes were transferred to solidified half-strength MS media, supplemented with 1% (w/v) sucrose and allowed to grow for another 10 days. Error bars represent standard errors (se), *n* ≥ 23. Letters depict significance in a one-way analysis of variance (ANOVA) followed by Tukey’s test (a and b, *P* < 0.001). The experiment was repeated twice with similar results. C, CE–ESI–MS analysis of inositol polyphosphate levels of 2-week-old Arabidopsis wild-type (Col-0), *itpk1* and complemented *itpk1* seedlings as indicated. Plants were grown on solidified half-strength MS media, supplemented with 1% (w/v) sucrose, growth conditions are detailed in the methods section. InsP species are presented as percentage of total InsP_6_. Data are means ± se (*n* = 6, biological replicates). Different letters indicate significance in one-way ANOVA followed by Tukey’s test (*P* < 0.05). D, Inositol polyphosphate enrichment from indicated plant extracts by TiO_2_ pull down. Inositol polyphosphates were eluted from TiO_2_ beads, separated by PAGE, and visualized by toluidine blue.

Compared to Col-0 wild-type, *itpk1* plants display a primary root elongation defect when grown on solidified half-strength Murashige and Skoog (MS) media (Figure 1, A and B). This short-root phenotype was rescued by introducing a genomic *ITPK1* construct containing an *ITPK1* promoter fragment and the *ITPK1* gene in translational fusion with C-terminal G3GFP (Figure 1, A and B; Supplemental Figure S1, A and B). The ITPK1–G3GFP fusion also rescued yeast *kcs1*Δ-associated growth defects (Supplemental Figure S1C), indicating that the InsP_6_-kinase activity reported for the native protein (Laha et al., 2019) remains intact in the translational fusion protein. We performed strong anion exchange (SAX)–high-performance liquid chromatography (HPLC) analyses of [^3^H]-*myo*-inositol-labeled seedlings grown in liquid half-strength MS media. Those analyses revealed a strong increase in as yet unknown isomers of InsP_3_ and InsP_4_, as well as reduced InsP_5_ [1/3-OH] and InsP_7_ levels in *itpk1* plants (Supplemental Figure S2, A and B). Next, extracts of unlabeled seedlings purified by TiO_2_ pull down were subjected to capillary electrophoresis coupled to electrospray ionization mass spectrometry (CE–ESI–MS). This method enables a sensitive discrimination and quantification of several symmetric and unsymmetric inositol polyphosphates, including all possible InsP_7_ isomers (Qiu et al., 2020; Riemer et al., 2021), without providing information about their enantiomeric identity. As depicted in Figure 1C, *itpk1* seedlings displayed an increase in two InsP_3_ and one InsP_4_ species of unknown structural identity and showed reduced InsP_5_ [1/3-OH] levels, in agreement with our SAX–HPLC data. CE–ESI–MS analyses further showed that *itpk1* seedlings have a two-fold decrease in 5-InsP_7_, a mild increase in 1/3-InsP_7_ and no change in 4/6-InsP_7_ (Figure 1C), a PP-InsP isomer recently identified in plants (Riemer et al., 2021). Other inositol polyphosphate species were not affected in *itpk1* plants (Figure 1C). These observations largely recapitulated *itpk1*-associated changes in inositol polyphosphates of older plants cultivated in an aerated hydroponic culture with sufficient supply of all nutrients (Riemer et al., 2021). Notably, no changes in InsP_6_ were observed between *itpk1* and Col-0 wild-type plants by either polyacrylamide gel electrophoresis (PAGE) analyses of TiO_2_ pull-down-purified extracts or by SAX–HPLC analyses (Figure 1D; Supplemental Figure S2, A and B). Importantly, all *itpk1*-associated inositol polyphosphate defects were fully rescued in complemented *itpk1* plants expressing the *ITPK1–G3GFP* fusion (Figure 1C).

### ITPK1 plays a critical role in auxin-related processes

**Figure 2:**
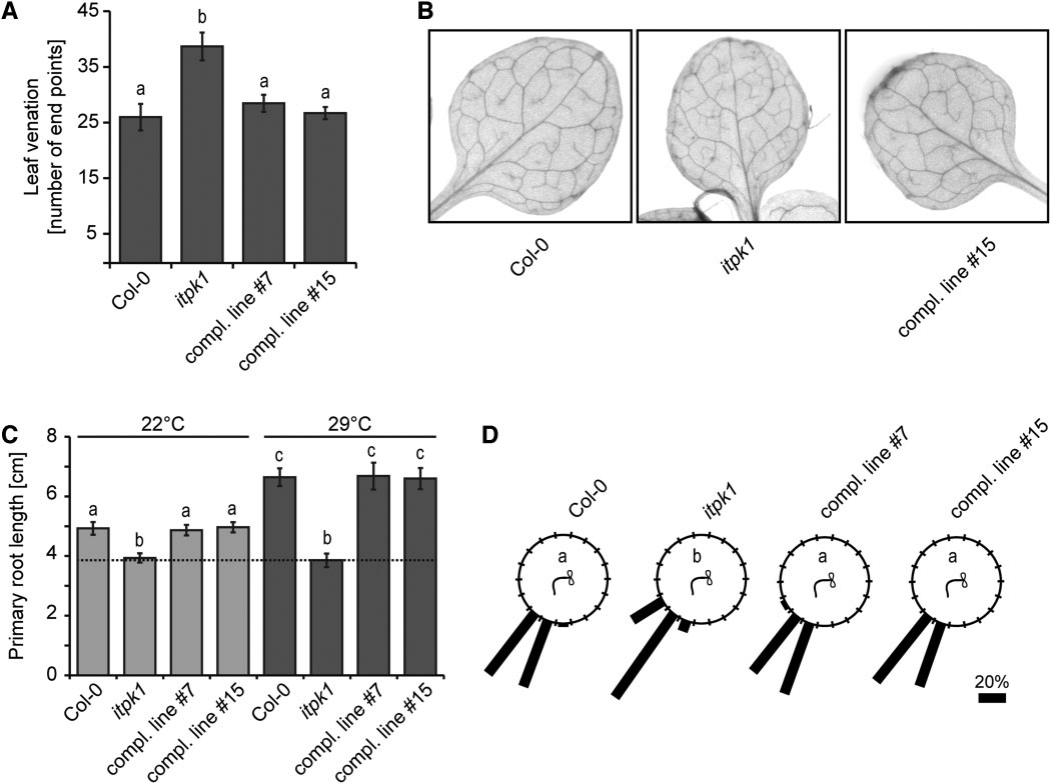
Leaf venation, root gravitropic, and thermomorphogenic responses are controlled by ITPK1. A and B, Analysis of leaf vasculature (A). Venation patterns were recorded from 13-day-old seedlings of the indicated genotypes grown on solidified half-strength MS media, supplemented with 1% (w/v) sucrose. Photographs of representative leaves of each genotype used to analyze venation patterns (B). Error bars depict sem (*n* = 6–10, biological replicates). The experiment was repeated twice with similar results. Letters depict significance in one-way ANOVA followed by Dunnett’s test (a and b, *P* < 0.005). C, Primary root length analysis of designated genotypes grown at different temperatures (C). Five-day-old seedlings were kept at 22°C or shifted to 29°C. Root length was evaluated after 8 days by ImageJ. Error bars represent sem (*n* ≥ 17, biological replicates). Letters depict significance in one-way ANOVA followed by Dunnett’s test (a and b, *P* < 0.005; a to c, *P* < 0.001; b to c, *P* < 0.001). The experiment was repeated independently with similar results. D, Root gravitropism of 12-day-old seedlings grown on solidified half-strength MS media, supplemented with 1% (w/v) sucrose. Indicated genotypes were rotated by 90° and gravitropic curvatures were measured after 16 h. The percentage of seedlings in each category are represented by the length of the bar. The distribution of data was analyzed using χ^2^ test (number of seedlings, *n* ≥ 36, groups contained at least 7.5% (w/v) of total seedlings per genotype). Significant differences (*P* <0.0001) are indicated by different letters. The experiment was performed independently with similar results.

Besides the short-root phenotype, we also noticed other developmental defects in the *itpk1* mutant. For instance, *itpk1* plants displayed an increased number of end points of the leaf vasculature, a defect that was rescued by expressing the *ITPK1–G3GFP* fusion (Figure 2, A and B). Because leaf venation and primary root architecture are regulated by auxin (Mattsson et al., 1999; Sieburth, 1999; Scarpella et al., 2006; Benjamins and Scheres, 2008), we analyzed other auxin-related processes. We first examined thermomorphogenesis, an adaptation response to elevated temperatures that is also regulated by auxin (Ruegger et al., 1998; Quint et al., 2016; Wang et al., 2016; Bellstaedt et al., 2019). As reported earlier, exposure of wild-type seedlings to 29°C resulted in increased primary root length (Wang et al., 2016). However, this high temperature-induced response was severely compromised in *itpk1* plants, whose primary root lengths remained unchanged upon the elevated temperature treatment (Figure 2C; Supplemental Figure S3A), resembling auxin receptor mutants (Wang et al., 2016). The compromised root development of *itpk1* plants under high temperature was again fully restored in complemented lines (Figure 2C; Supplemental Figure S3A). We further noticed that ITPK1-deficient plants are also defective in gravitropic root curvature. This defect was fully rescued in *itpk1* plants expressing genomic *ITPK1* (Figure 2D).

**Figure 3:**
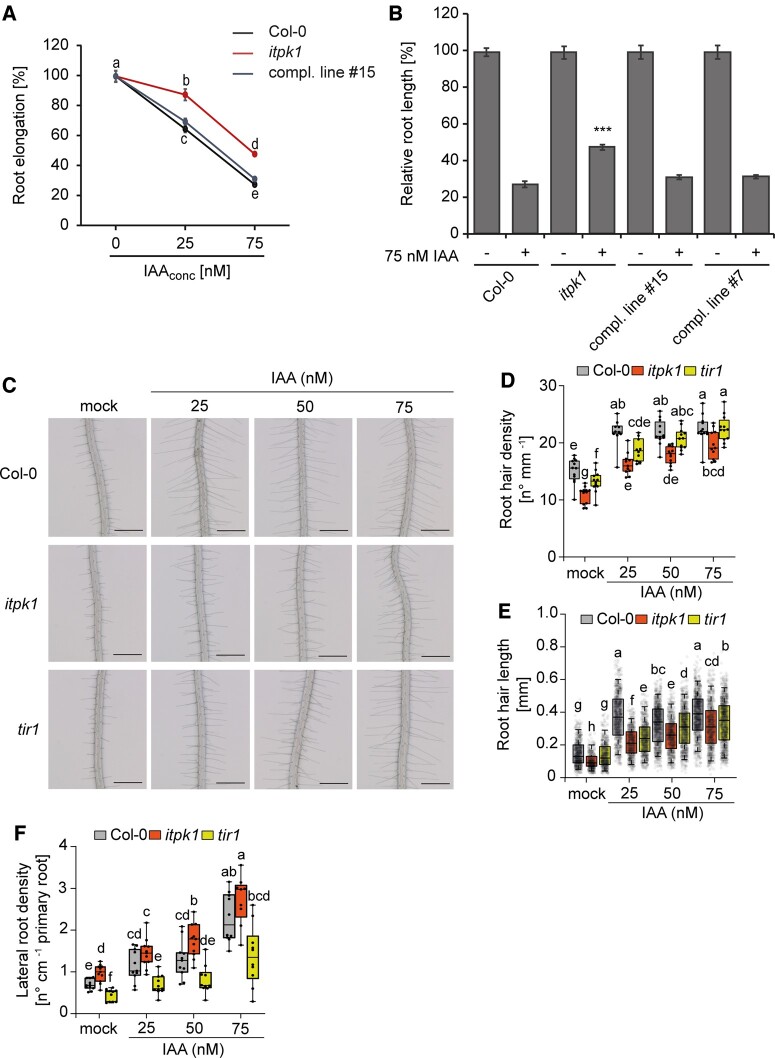
Loss of ITPK1 results in compromised auxin responses. A, Relative root elongation of designated genotypes under increasing IAA concentrations. Six-day-old seedlings were transferred to solidified half-strength MS media, supplemented with 1% (w/v) sucrose and 0, 25, and 75-nM IAA as indicated and incubated for 5 days. Root lengths were evaluated by ImageJ. Error bars are sem (*n*=10–35). Different letters indicate significance in one-way ANOVA followed by Dunnett’s test (a and b, *P* < 0.05; a to c, *P* < 0.001; a to d, *P* < 0.001; b to c, *P* < 0.001; d and e, *P* < 0.001; b to d, *P* < 0.001; c to e, *P* < 0.001). The experiment was repeated twice with similar results. B, Relative root length of wild-type (Col-0), *itpk1* mutant and selected complemented lines treated with 75-nM IAA. Six-day-old seedlings of designated genotypes were transferred to solidified half-strength MS media, supplemented with 1% (w/v) sucrose and 0.75-nM IAA. After 7-days of growth, images were taken and root lengths were evaluated by ImageJ. Error bars present sem (*n* = 10–35). Asterisks indicate statistical differences (treated wild-type versus all treated all other genotypes, Student’s *t* test; ***P < 0.0001). C–E, Root hair formation of designated genotypes under increasing IAA concentrations. Six-day-old seedlings were transferred to solidified [0.8% (w/v) phytagel], half-strength MS media supplemented with 1% (w/v) sucrose and with 0, 25, 50, or 75 nM IAA as indicated and incubated for 5 days. Representative root images (C) and quantifications of root hair density (D) and root hair length (E). Root hair lengths were evaluated by ImageJ. Boxplots show the first quartile, median and third quartile. The whiskers extend to the minimum and maximum values (D) with *n* = 10–12 independent roots, or to 5th and 95th percentile (E) with *n* = 457–951 independent root hairs from 10–12 roots. Different letters indicate significance in one-way ANOVA followed by Tukey’s test (*P* < 0.05). Scale bars, 500 µm. F, Lateral root density of designated genotypes under increasing IAA concentrations. Six-day-old seedlings were transferred to solidified half-strength MS media supplemented with 1% (w/v) sucrose and 0, 25, 50, or 75 nM IAA as indicated and incubated for 6 days. Boxplots show the first quartile, median and third quartile. The whiskers extend to the minimum and maximum values with *n* = 9–11 independent roots. Different letters indicate significance in one-way ANOVA followed by Tukey’s test (*P* < 0.05).

The phenotypic defects exhibited by *itpk1* plants are reminiscent of impaired auxin signaling (Sieburth, 1999; Scarpella et al., 2006; Teale et al., 2006; Salehin et al., 2015; Weijers and Wagner, 2016). To further test this possibility, we performed auxin sensitivity assays (Lincoln et al., 1990; Ruegger et al., 1998) by determining the root length after exogenous application of the natural auxin indole-3-acetic acid (IAA). Wild-type seedlings displayed gradually shorter roots in the presence of increasing amounts of IAA. On the other hand, *itpk1* seedlings were more resistant to the exogenous supply of auxin, as root growth in these plants was not as severely affected by treatment with the synthetic auxin 1-naphthaleneacetic acid (NAA) as in the wild-type (Figure 3, A and B; Supplemental Figure S3B). Auxin-insensitivity of *itpk1* plants was fully rescued in independent complemented lines expressing the genomic *ITPK1* fragment (Figure 3, A and B). In order to assess whether *itpk1* plants are impaired in another auxin-dependent process, we then evaluated root hair formation in response to increasing exogenous IAA concentrations. Overall, the length and density of root hairs were significantly altered in *itpk1* mutant plants, partially resembling the auxin–receptor mutant *tir1* (Figure 3, C–E). Although root hair formation was stimulated by auxin, *itpk1* plants exhibited fewer and shorter root hairs than wild-type plants even when supplied with 75-nM IAA. With these observations, we concluded that ITPK1 has an important function in various auxin-related growth and developmental processes.

We then also investigated the lateral root development both in wild-type and *itpk1* knockout plants (Figure 3F). However, unlike root hair and primary root development, auxin-induced lateral root formation was not significantly different between wild-type and *itpk1* plants under most exogenous IAA concentrations, suggesting that lateral root development may not to be regulated by ITPK1 activity.

### Impaired auxin responses in *itpk1* plants are not caused by altered auxin synthesis or transport

**Figure 4:**
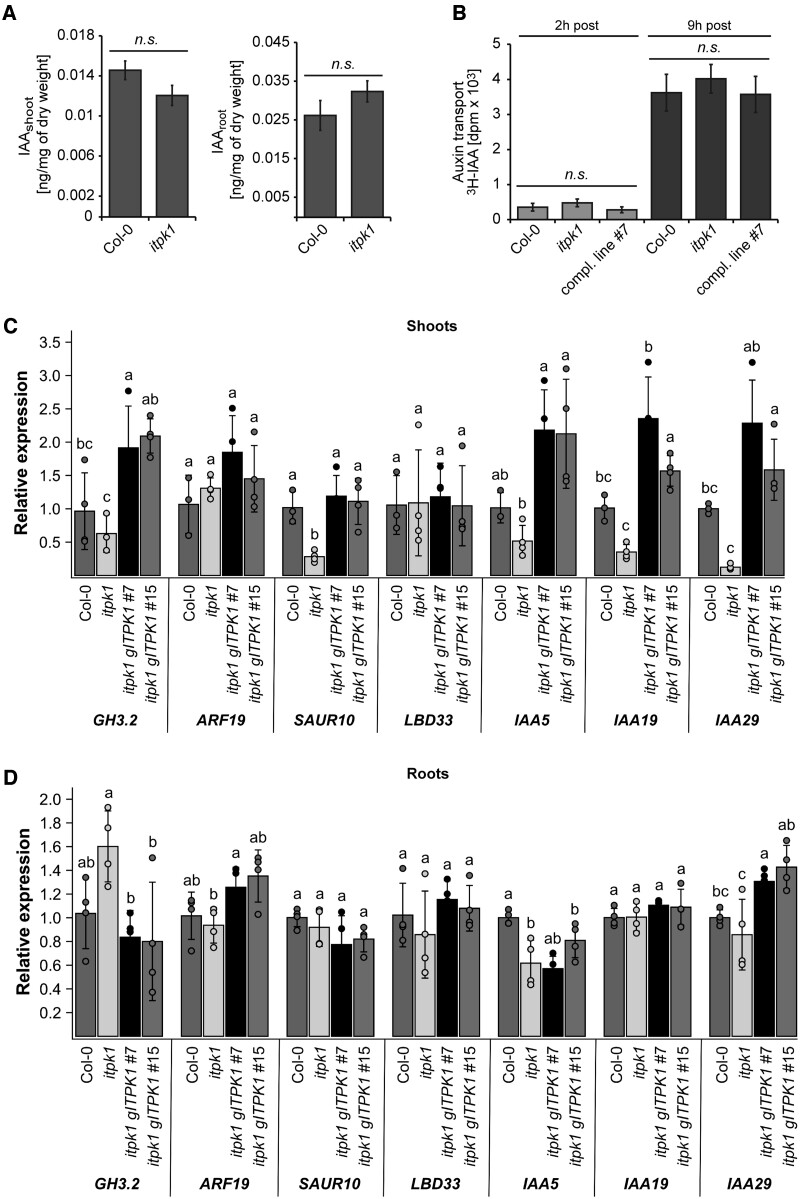
Arabidopsis *itpk1* plants are not defective in auxin synthesis and transport but are compromised in auxin-related gene expression. A, Auxin (IAA) levels in shoot and roots of 2-week-old seedlings of designated genotypes grown on solidified half-strength MS media, supplemented with 1% (w/v) sucrose. Error bars represent sem (*n* ≥ 7, biological replicates). The experiment was repeated with similar results. Statistical significance was determined by two-tailed Student’s *t* test (*P* < 0.01). Differences between the designated genotypes were not significant (n.s.) as indicated. B, Polar auxin transport. The apical end of excised stems of designated genotypes were placed in liquid solidified half-strength MS media, supplemented with 1% (w/v) sucrose and [^3^H] IAA. After indicated times of incubation, the basal ends of the labelled stems were excised, and the activity was determined by scintillation counting. Error bars represent sem (*n* = 3, biological replicates). Statistical significance was determined by two-tailed Student’s *t* test (*P* < 0.01). Genotypes in all panels are as indicated. Differences between the designated genotypes were not significant (n.s.) as indicated. C and D, Relative expression of auxin-responsive genes in shoot (C) and root (D) of wild-type (Col-0), *itpk1* mutant and two selected complemented lines, determined by RT–qPCR analyses using RNA extracted from 2-week-old seedlings grown on solidified half-strength MS media, supplemented with 1% (w/v) sucrose. *Βeta-TUBULIN* served as a reference gene. Shown are means ± sd (*n* = 3 or 4). Letters indicate significant differences between the lines in each gene by one-way ANOVA and Tukey’s HSD test (*P* < 0.05).

We then asked whether ITPK1 affects auxin synthesis or transport, by evaluating auxin levels and polar auxin transport in wild-type and *itpk1* plants. As depicted in Figure 4, A and B, auxin levels in shoots and roots, as well as polar auxin transport were similar between *itpk1* and Col-0 wild-type plants, suggesting that the observed phenotypic differences were not related to either the synthesis or the transport of auxin. We also tested sensitivity to the synthetic auxin analog NAA, which can bypass auxin carrier-mediated transport mechanisms, as it diffuses more easily through membranes than IAA (Delbarre et al., 1996). The *itpk1* line was also less sensitive to NAA with respect to primary root elongation as compared to wild-type plants (Supplemental Figure S3B), further supporting that altered auxin transport is unlikely to cause the observed phenotypes. In contrast, the expression of several marker genes associated with auxin signaling were compromised in *itpk1* plants and rescued in complemented lines, an effect that was more pronounced in shoots than in roots (Figure 4, C and D).

### Defects in phosphate homeostasis and in auxin responses are largely independent consequences of *itpk1* loss of function

Recently, it was reported that *itpk1* plants exhibit constitutive phosphate starvation responses, which result in phosphate over-accumulation in leaves when plants are grown with sufficient phosphate supply (Kuo et al., 2018; Riemer et al., 2021). To assess whether increased phosphate accumulation can affect auxin sensitivity, we analyzed the *pho2* mutant, in which disrupted phosphate signaling downstream of PHR1 leads to phosphate over-accumulation (Aung et al., 2006; Bari et al., 2006). Notably, we did not find significant differences between wild-type and *pho2-1* plants with respect to gravitropic responses, sensitivity of primary root growth to auxin or high temperature-induced elongation of primary roots (Supplemental Figure S4, A–C). We also found that the over-accumulation of phosphorus in *itpk1* plants was largely unaffected by auxin (Supplemental Figure S5A), suggesting that exogenously applied auxin does not alter phosphate accumulation in shoots. However, when plants were grown on low phosphate, a condition that strongly inhibits primary root elongation (Gutierrez-Alanis et al., 2018), the auxin insensitive primary root growth of *itpk1* plants was not observed anymore (Supplemental Figure S5, B and C). Overall, these findings indicate that the impaired phosphate starvation responses of *itpk1* plants under sufficient phosphate supply does not explain the auxin-related phenotypes and that both uncontrolled phosphate accumulation and defective auxin responsiveness are independent consequences of impaired ITPK1 activity.

### Defects in auxin responses correspond to changes in InsP and PP-InsP homeostasis

To dissect which inositol derivatives are possibly involved in auxin signaling, we employed different mutants affected in PP-InsP and InsP synthesis. To address the question of whether InsP_8_ might be involved in auxin perception, we investigated two independent *vih2* lines (*vih2-3* and *vih2-4*) that have strongly reduced InsP_8_ levels in vegetative tissues (Laha et al., 2015; Riemer et al., 2021). Notably, both mutants were undistinguishable from Col-0 wild-type plants with respect to thermomorphogenesis, gravitropism, and sensitivity of the primary root to auxin (Supplemental Figure S6, A–C). These results suggest that bulk InsP_8_ has no critical role in auxin signaling. In contrast, similar to *itpk1*, auxin responses were also significantly decreased in plants defective in the InsP_5_ [2-OH] kinase IPK1 (Supplemental Figure S7, A and B). Respective *ipk1-1* mutant plants display compromised conversion of InsP_5_ [2-OH] to InsP_6_ (Stevenson-Paulik et al., 2005; Kuo et al., 2014) and hence have reduced InsP_6_, InsP_7_, and InsP_8_ levels (Laha et al., 2015). These findings were analyzed in more detail by CE–ESI–MS measurements that show *ipk1* plants are defective in one InsP_3_ and InsP_4_ species, InsP_5_ [1/3-OH], InsP_5_ [4/6-OH], 5-InsP_7_, as well as 1/3-, and 4/6-InsP_7_, while they accumulate more of one InsP_3_ and one InsP_4_ species of unknown structural identity (Supplemental Figure S7C).

Thus, common denominators between *ipk1-1* and *itpk1* mutant plants are decreases in InsP_5_ [1/3-OH] and 5-InsP_7_, and an increase in InsP_3_-3 and InsP_4_-1 (Figure 1C; Supplemental Figures S2 and S7C), largely corroborating earlier findings (Stevenson-Paulik et al., 2005; Laha et al., 2015; Riemer et al., 2021), and suggesting that one or several of these InsP isomers are critical for auxin signaling.

### In vitro reconstitution assays suggest that highly anionic inositol polyphosphates bind to the auxin–receptor complex with high affinity

Considering the unknown isomer identity of the InsP_4_ species accumulating in *itpk1* lines (15 distinct InsP_4_ isomers are possible but only a few are commercially available, which allows limited evaluation of these isomers), we purified the InsP_4a_ species from the *itpk1* SAX-HPLC run. This species most likely represents InsP_4-1_ detected by CE–ESI–MS analyses. When incubated with recombinant ITPK1 and ATP, purified InsP_4a_ was efficiently phosphorylated (Supplemental Figure S8A), suggesting that InsP_4a_ is an in vivo substrate of ITPK1. Furthermore, we performed direct auxin receptor complex binding assays with purified [^3^H]-InsP_3b_ (likely representing InsP_3-3_ detected by CE–ESI–MS), and [^3^H]-InsP_4a_ that accumulate in ITPK1-deficient plants and found that binding of the [^3^H]-InsP_4_ species is detectible, although less efficiently as compared to InsP_6_ (Supplemental Figure S8B). No detectible binding of the [^3^H]-InsP_3_ species accumulating in *itpk1* seedlings was observed (Supplemental Figure S8B). These data suggest that InsP_4a_ cannot be excluded as a potential direct (negative) regulator of auxin perception, while InsP_3b_ is unlikely to play a direct role in auxin receptor regulation.

**Figure 5:**
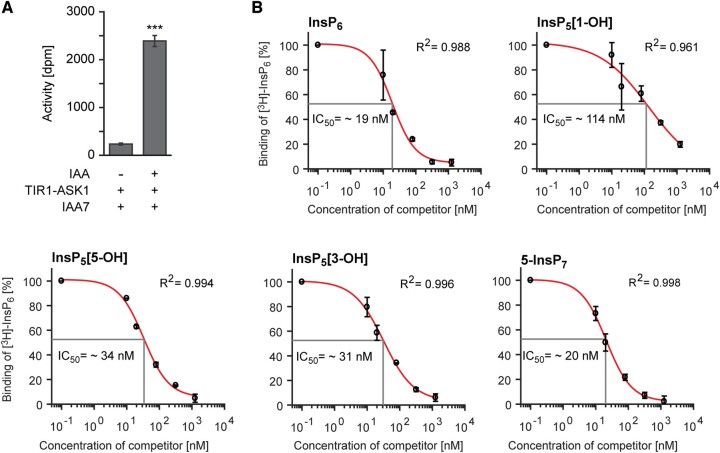
Highly anionic inositol polyphosphates bind to the auxin receptor complex with high affinities. A, Auxin-dependent binding of [^3^H]-InsP_6_ to the TIR1–ASK1–IAA7 complex. Recombinant His_8_-MBP-IAA7 was incubated with insect cell-purified TIR1–ASK1 and [^3^H]-InsP_6_ in the presence of 0.1-μM IAA. A reaction in the absence of auxin served as a negative control. Values show background-subtracted means ± se (*n* = 3), ***P < 0.001, two-tailed Student’s *t* test. B, Concentration-dependent competitive binding of [^3^H]-InsP_6_ to the ASK1–TIR1–Aux/IAA–IAA receptor complex in the presence of different unlabeled inositol polyphosphate species. Results are presented as the percentage of total binding. To determine IC_50_ values, nonlinear regression analyses were employed to fit data to a sigmoidal model. *R*^2^ values presented in the plots provide estimations for goodness of fit. Error bars represent se (*n* = 2).

To further investigate the contribution of ITPK1 in auxin perception, we performed competitive binding assays with dialyzed insect cell-purified ASK1–TIR1, recombinant Aux/IAA protein, natural auxin (IAA), and [^3^H]-InsP_6_ to determine IC_50_ values (50% displacement of radioligand binding) for ITPK1-dependent InsPs and related molecules. We used His_8_-tagged recombinant Aux/IAA7 protein to pull down ASK1–TIR1 in the presence of auxin via Ni-nitrilotriacetic acid (Ni–NTA) affinity chromatography. Very little [^3^H]-InsP_6_-derived activity could be recovered in the pull-down assay when auxin was not included in the reaction (Figure 5A), demonstrating that auxin is critical for the interaction between ASK–TIR1 and Aux/IAA protein. IC_50_ values of different InsP species to compete with [^3^H]-InsP_6_ were as follows: InsP_6_ (IC_50_: 19 nM) ≤ 5-InsP_7_ (IC_50_: 20 nM) < InsP_5_ [3-OH] (IC_50_: 31 nM) < InsP_5_ [5-OH] (IC_50_: 34 nM) < InsP_5_ [1-OH] (IC_50_: 114 nM) (Figure 5B). These results indicate that the ASK1–TIR1–IAA complex has distinct binding affinities towards different InsP isomers (including enantiomers) with InsP_6_ and 5-InsP_7_ displaying the highest affinities.

### ITPK1 interacts with TIR1

**Figure 6:**
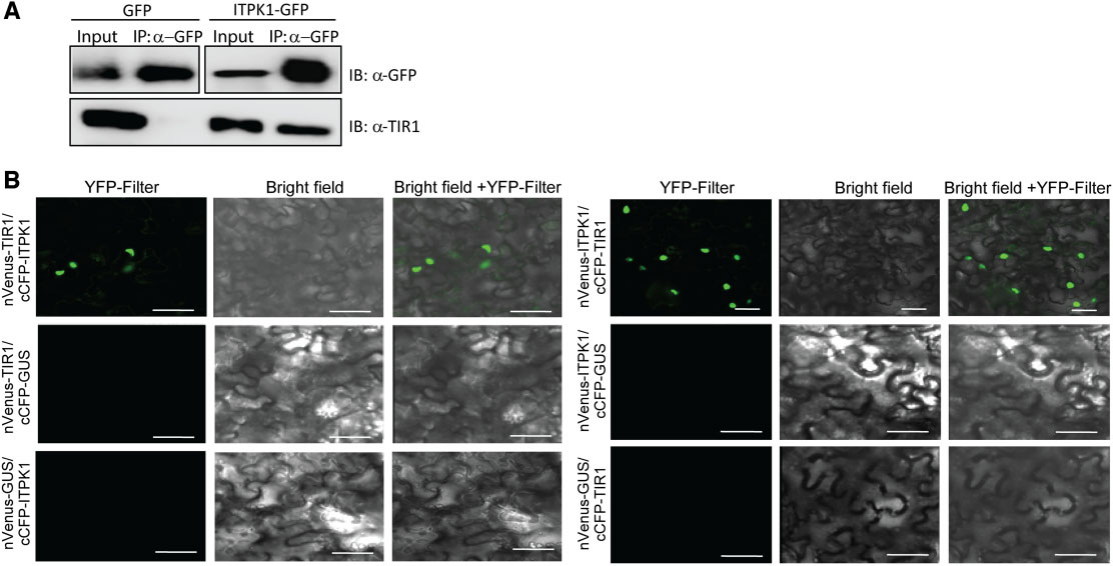
Arabidopsis ITPK1 physically interacts with TIR1. A, Enrichment of TIR1 from *ITPK1–G3GFP* expressing transgenic plants. Total extracts from 4-week-old ITPK1*–*G3GFP (compl. line#7) or from GFP alone expressing transgenic plants were immuno-enriched (IP) with anti-GFP-(right panel) conjugated agarose beads. Bound proteins and input extracts were immunoblotted (IB) with anti-GFP (upper lanes) or anti-TIR1 (lower lanes) antibodies. B, Transiently expressed ITPK1 interacts with TIR1 in the nucleus of *N. benthamiana* cells. Combinations of co-expressed nVenus- or cCFP-tagged ITPK1, TIR1, or GUS (control) constructs are shown at the left of each panel. Images are presented as YFP fluorescence (YFP filter) and merge with bright field of the same section (brightfield + YFP filter). Scale bar = 50 µm.

A previously identified interaction of mammalian InsP_6_-kinase IP6K2 with a protein complex activated by casein kinase 2, an enzyme that requires InsP_7_ for full activity (Rao et al., 2014), suggested that, in mammalian cells, PP-InsPs might be generated in close proximity to dedicated effector proteins. Since 5-InsP_7_, an ITPK1-dependent PP-InsP, binds with strong affinity to the TIR1–ASK1 receptor complex (Figure 5B), we investigated whether ITPK1 physically interacts in planta with TIR1 to facilitate targeted delivery of 5-InsP_7_. To test this hypothesis, we first performed co-immunoprecipitation assays. The Arabidopsis *itpk1* line complemented with the *gITPK1-G3GFP* (compl. line#7) or a transgenic line expressing GFP alone (as a negative control) were used for immuno-enrichment with anti-GFP antibodies and probed for the presence of TIR1 in the co-elute with anti-TIR1 antibodies. ITPK1*–*G3GFP, but not GFP alone, allowed detection of TIR1, suggesting that ITPK1 associates with TIR1 in vivo (Figure 6A).

To further validate the identified interaction between ITPK1 and TIR1, we next used bimolecular fluorescence complementation (BiFC) assays. Co-expression of nVenus-ITPK1 with cCFP-TIR1 or the reciprocal combination in *Nicotiana benthamiana* leaves resulted in reconstituted YFP fluorescence detectable in the nucleus of transformed cells (Figure 6B). In contrast, no fluorescence was detected when BiFC construct of GUS was expressed either with ITPK1 or TIR1, reinforcing the specificity of ITPK1–TIR1 association in these assays (Figure 6B). Taken together, these results demonstrate that ITPK1 physically interacts with TIR1 in planta.

### ITPK1 and ITPK2 function redundantly to control auxin responses

**Figure 7:**
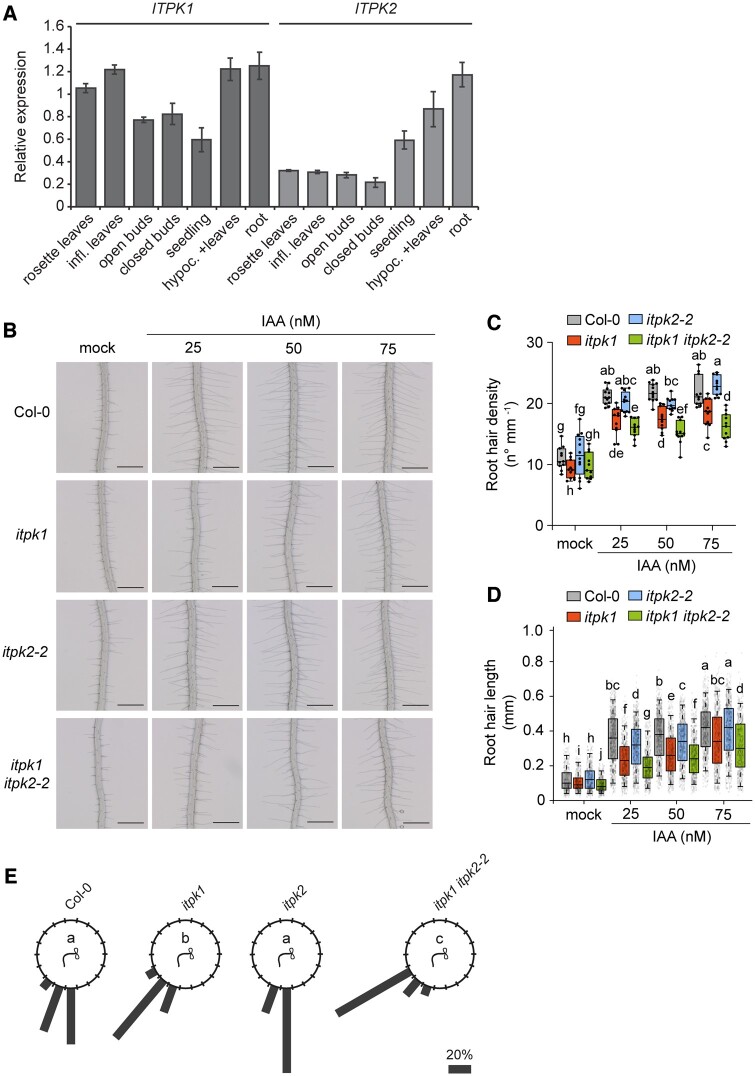
ITPK1 and ITPK2 function redundantly to control auxin responses. A, Expression analyses of Arabidopsis *ITPK1* and *ITPK2*. RT–qPCR analyses of *ITPK1* and *ITPK2* using cDNA prepared from RNA extracts of different tissues of wild-type Col-0 plants. Infl. refers to inflorescence, hypoc. refers to hypocotyl. Error bars represent standard deviation (sd, *n* = 3 technical replicates). *PP2AA3* was used as reference gene. The experiment was repeated independently with similar results. B–D, Representative root images (B) and quantifications of root hair density (C) and root hair length (D) under increasing exogenous IAA application. Six-day-old seedlings were transferred to solidified [0.8% (w/v) phytagel], half-strength MS media supplemented with 1% (w/v) sucrose and with 0, 25, 50, and 75 nM IAA as indicated and incubated for 5 days. Root hair lengths were evaluated by ImageJ. Boxplots show the first quartile, median, and third quartile. The whiskers extend to the minimum and maximum values (C) with *n* = 11–12 independent roots, or to 5th and 95th percentile (D) with *n* = 411–923 independent root hairs from 11–12 roots. Different letters indicate significance in one-way ANOVA followed by Tukey’s test (*P* < 0.05). Scale bars, 500 µm. E, Root gravitropism of seedlings of wild-type (Col-0), *itpk1, itpk2-2*, and *itpk1 itpk2-2* after 90° reorientation. Six-day-old seedlings were transferred to fresh solidified half-strength MS media, supplemented with 1% (w/v) sucrose and after another 7 days of growth, the seedlings of indicated genotypes were rotated by 90° and the gravitropic curvature was measured after 16 h. The percentage of seedlings in each category are represented by the length of the bar. The distribution of data was analyzed using a χ^2^ test (number of seedlings, *n* ≥ 11). Means with different letters are significantly different, *P* < 0.001.

The discovery that ITPK2 displays InsP_6_-kinase activity in vitro similar to ITPK1 (Adepoju et al., 2019; Laha et al., 2019) encouraged us to investigate whether both homologs act redundantly in InsP/PP-InsP homeostasis and in the regulation of auxin-related processes. Reverse transcription–quantitative polymerase chain reaction (RT–qPCR) analyses revealed ubiquitous expression of both homologs (Figure 7A). While high levels of *ITPK1* transcripts were detected in all plant tissues, the expression of *ITPK2* was notably strong in the root and hypocotyl. However, the disruption of *ITPK2* did not result in any obvious InsP/PP-InsP defect, as deduced from the largely similar SAX-HPLC profiles of *itpk2-2* mutant lines and Col-0 (Supplemental Figure S9A). This was in agreement with previous CE–ESI–MS analyses that did not reveal any InsP/PP-InsP defects of *itpk2-2* seedlings grown under P_i_-replete conditions (Riemer et al., 2021). The absence of InsP/PP-InsP alterations might also explain why *itpk2-2* plants did not display any defects in auxin-dependent processes (Figure 7, B–E; Supplemental Figure S9, B and C). However, despite the different phenotypes of *itpk1* and *itpk2-2* mutants, a possible functional redundancy might still explain why *itpk1* plants do not display auxin perception defects as severe as higher order auxin–receptor knockout lines (Prigge et al., 2020). We therefore analyzed auxin responses of a recently isolated *itpk1 itpk2-2* double mutant (Riemer et al., 2021). We found that both root hair density and root hair length were more severely perturbed in *itpk1 itpk2-2* plants as compared to the *itpk1* single mutant (Figure 7, B–D). Furthermore, root gravitropic responses were more strongly compromised in the *itpk1 itpk2-2* double mutant than in *itpk1* plants (Figure 7E; Supplemental Figure S10). Taken together, these results indicate that ITPK1 and ITPK2 act redundantly to control auxin responses in Arabidopsis.

## Discussion

The previously published crystal structure of the auxin receptor complex with insect-purified InsP_6_ (Tan et al., 2007) revealed a large hydrophilic binding pocket, in which InsP_6_ is coordinated by extensive interactions with basic residues of the concave surface of the TIR1 LRR solenoid (Supplemental Figure S11A; Tan et al., 2007). Reminiscent for what has been predicted for the InsP_8_-bound F-box component COI1 of the jasmonate receptor complex (Laha et al., 2016), these interactions are highly anisotropic. However, whether binding of InsP_6_ or any other inositol polyphosphate to auxin receptor complexes occurs in vivo, and whether such binding has physiological consequences for auxin perception, remained unresolved. Our work indicates that IPK1- and ITPK1-dependent InsPs/PP-InsPs are critical to fine-tune plant responses to auxin. The impaired auxin responses of *ipk1* and *itpk1* plants are most likely not caused by compromised stability of the auxin co-receptor F-box protein TIR1, as suggested by immunoblot analyses (Supplemental Figure S11B). Instead, we propose that IPK1- and ITPK1-derived InsPs/PP-InsPs activate the auxin receptor complex, therefore resulting in more efficient auxin signaling. As illustrated in Supplemental Figure S11A, basic residues distal and proximal to the hormone binding pocket likely contribute to the elliptical shape of the LRR solenoid. Notably, the 5-position of InsP_6_ is protruding toward the IAA7 degron residue R90 (dashed line). The distance of 5.6 Å between the 5-phosphate and closest amino group of the R90 side chain suggests that the pyrophosphate moiety of 5-InsP_7_, if oriented similarly as InsP_6_, would stabilize the IAA7 degron interaction with an additional strong polar interaction. Considering the architecture of the SCF^TIR1^ auxin receptor complex (Dinesh et al., 2016), even small changes in the orientation of the IAA degron relative to TIR1 are likely to have strong consequences with respect to the ubiquitination efficiency, and thus degradation of the Aux/IAA repressor and subsequent activation of auxin-responsive gene expression. Such 5-InsP_7_-dependent alterations in auxin receptor complex geometry might also help to discriminate different Aux/IAA transcriptional repressors, therefore not simply inducing a global auxin response, but rather result in a more complex and fine-tuned transcriptional regulation. This hypothesis potentially explains the differentiated transcriptional response observed in *itpk1* mutant plants (Figure 4, C and D). In the absence of a 5-InsP_7_-bound crystal structure of the auxin receptor complex, in silico docking experiments combined with molecular dynamics simulations and in parallel *cryo*-electron microscopy studies could provide more mechanistic details on the role of 5-InsP_7_ on auxin receptor function.

The concept that efficient auxin perception by the auxin receptor complex might require two ligands, that is auxin and 5-InsP_7_ (or another ITPK1-dependent InsP), appears reminiscent of jasmonate perception reported to rely on the coincidence detection of a JA-conjugate and InsP_8_ (Laha et al., 2015). Such coincidence detection might be used to fine-tune hormone response depending on other internal or external cues.

Our work reveals a physical interaction of ITPK1 with TIR1 (Figure 6). While further work will be necessary to establish whether this interaction is of functional relevance and how it is regulated, we hypothesize that the apparent close proximity of ITPK1 to the auxin receptor complex might create a privileged, local enrichment of InsPs/PP-InsPs to a dedicated effector protein complex, similar to what has been observed for casein kinase 2 in mammalian cells (Rao et al., 2014). While we cannot exclude the possibility that the interaction of ITPK1 and TIR1 might help to generate an important signaling molecule at minimal energetic costs, we speculate that this interaction more likely represents a mechanism to prevent or to control the stimulation of other signaling events triggered by the same molecule. This would be particularly intriguing for plant PP-InsPs, whose long distance cellular movement is likely restricted, assuming their lifetime is similarly short as mammalian PP-InsPs (Menniti et al., 1993).

To better understand the role of 5-InsP_7_ and other ITPK1-derived InsPs/PP-InsPs in auxin perception, it will be important to examine whether also AFBs interact with ITPK1 or other InsP kinases. It will also be important to know whether such dedicated interactions, and hence localized generation of regulatory molecules, might contribute to the specificity by which auxin controls many different aspects of plant growth and development. To explore if and how InsPs and PP-InsPs can contribute to the crosstalk between nutritional cues and jasmonate and auxin signaling, tools have to be developed to follow InsPs and PP-InsPs with high specificity and with high temporal and spatial resolution. The work presented here provides an important step to unveil the physiological processes regulated by 5-InsP_7_ and other ITPK1-dependent inositol polyphosphates and opens avenues to manipulate and better understand PP-InsP signaling in eukaryotic cells.

## Materials and methods

### Plant material and growth conditions

Seeds of T-DNA insertion lines of Arabidopsis (*A. thaliana*; ecotype Col-0) were obtained from The European Arabidopsis Stock Center (http://arabidopsis.info/). The *itpk1* (SAIL_65_D03) and *itpk2-2* (SAIL_1182_E03) insertion lines were genotyped for homozygosity using T-DNA left and right border primers and gene-specific sense or antisense primers (Supplemental Table S1). The *pITPK1:gITPK1–G3GFP* construct was generated as described in the cloning section. Auxin assays were performed with *tir1* [*tir1-1* allele; (Ruegger et al., 1998)], *vih2-3*, *vih2-4*, and *ipk1-1* (Laha et al., 2015), *itpk1 itpk2-2* (Riemer et al., 2021), as well as *pho2-1* (Delhaize and Randall, 1995) mutant plants. Wild-type Col-0 and all relevant transgenic lines were amplified together on a peat-based substrate (GS90) under identical conditions (16-h light and 8-h dark, day/night temperatures 22/18°C and 120 µmol^−1^ m^−2^ light intensity), and seeds of the respective last progenies were harvested and used for all analyses described in this article. For sterile growth, seeds were surface sterilized in 70% (v/v) ethanol and 0.05% (v/v) Triton X-100 for 30 min and washed twice with 90% (v/v) ethanol. Sterilized seeds were sown on solidified half-strength MS media [0.8% (w/v) phytagel], supplemented with 1% (w/v) sucrose, were stratified for 2 days at 4°C, and grown under conditions of 8-h light (23°C) and 16-h dark (21°C), unless mentioned otherwise.

### Constructs and strains

The *IAA7* open reading frame (ORF) was amplified from cDNA of 2-week-old wild-type Col-0 seedlings. The forward and reverse primes used to amplify the ORFs contained restriction sites as indicated in Supplemental Table S1. Amplified PCR products were inserted into CloneJET (Thermo Scientific) following the manufacturer’s instructions. The ORFs were then excised from respective CloneJET vectors and subcloned into the pET28-His_8_-MBP vector (Laha et al., 2015).

For complementation of *itpk1* plants, a genomic fragment encoding ITPK1 including a 1,839-bp region upstream of the *ITPK1* start codon was amplified from wild-type Col-0 genomic DNA, cloned into pENTR-D-TOPO and recombined with pGWB550 (Nakagawa et al., 2007) to generate a plant transformation vector containing the genomic *ITPK1* in translational fusion with a C-terminal G3GFP. Transformed lines were selected on solid plant media with 25-µg mL^−1^ hygromycin. For BiFC constructs, *ITPK1* and *TIR1* sequences were amplified from total cDNA from wild-type Col-0 plants. The primer sequences are shown in Supplemental Table S1. The amplicons were first cloned into the entry vector pDONR207 (Thermofisher) using BP Clonase (Invitrogen) and subsequently recombined using Clonase LR (Invitrogen) into the destination vectors pMDC-nVenus or pMDC-cCFP (Bhattacharjee et al., 2011). The pENTR-GUS plasmid (Invitrogen) was similarly cloned into the BiFC destination vectors pMDC-nVenus or pMDC-cCFP. Resulting clones were confirmed by sequencing. The binary vectors were electroporated into the agrobacterium (*Agrobacterium tumefaciens*) strain GV3101, and used for BiFC assays.

### Extraction and SAX–HPLC analyses of yeast and *A. thaliana*

Extraction and quantification of InsPs from yeast and from Arabidopsis seedlings were carried out as described (Laha et al., 2015). For the latter, 10-day-old Arabidopsis seedlings grown in 8-h light/16-h dark condition on solidified half-strength MS media, supplemented with 1% (w/v) sucrose, were transferred to 3-mL liquid sterile media containing half-strength MS media, supplemented with 1% (w/v) sucrose and 30 μCi mL^−1^ of [^3^H]-*myo*-inositol (30–80 Ci mmol^−1^; Biotrend; ART-0261-5). Seedlings were allowed to label for 6 days, then washed twice in dH_2_O and frozen in liquid N_2_. Extraction of InsPs was done as described (Azevedo and Saiardi, 2006; Laha et al., 2021), and extracts were resolved by SAX–HPLC using a Partisphere SAX 4.6 × 125 mm column (Whatman) at a flow rate of 0.5 mL min^−1^ with a shallow gradient formed by buffers A (1-mM EDTA) and B [1-mM EDTA and 1.3-M (NH_4_)_2_HPO_4_, pH 3.8, with H_3_PO_4_] (Laha et al., 2015; Gaugler et al., 2020).

### CE–ESI–MS measurements

InsP extracts from TiO_2_ purification were dissolved in 30-µL water. Five microliters of InsP extracts were mixed with 5-µL isotopic standards mixture (4-µM [^13^C_6_]1,5-InsP_8_, 4-µM [^13^C_6_] 5-InsP_7_, 4-µM [^13^C_6_] 1-InsP_7_, 40-µM [^13^C_6_] InsP_6_, and 8-µM [^13^C_6_] 2-OH InsP_5_) for CE–ESI–MS measurements (Harmel et al., 2019; Puschmann et al., 2019).

The CE–ESI–MS analysis of inositol polyphosphates in planta has been recently described (Riemer et al., 2021). The CE–ESI–MS for this study was performed on the same setup, which is an Agilent 7100 CE coupled with a triple quadrupole tandem mass spectrometry Agilent 6495c, equipped with an Agilent Jet Stream ESI source and CE–MS sheath liquid coaxial interface. All experiments were performed on a fused silica capillary with a length of 100 cm (50-μm internal diameter and 365-μm outer diameter). About 40-mM ammonium acetate titrated by ammonia solution to pH 9.0 was employed as background electrolyte. Samples were injected by applying 100 mbar pressure for 10 s, corresponding to 1% of the total capillary volume (20 nL). The separation voltage was 30 kV.

The sheath liquid was a mixture of water–isopropanol (1/1, v/v), which was introduced at 10 µL min^−1^. The MS source parameters settings were as follows: nebulizer pressure was 8 psi, gas temperature was 150°C and with a flow of 11 L min^−1^, sheath gas temperature was 175°C and with a flow at 8 L min^−1^, the capillary voltage was −2,000 V with nozzle voltage 2,000 V. Negative high-pressure radio frequency (RF) and low-pressure RF (Ion Funnel parameters) was 70 V and 40 V, respectively. Mass spectrometer parameters for multiple reaction monitoring (MRM) transitions are shown below. MRM parameter settings for InsPs and PP-InsPs are detailed in Supplemental Table S2.

### Root gravitropism assays

Seedlings were grown on vertical plates containing solidified half-strength MS media, supplemented with 1% (w/v) sucrose under 8-h light/16-h dark condition. After 7 days, seedlings were transferred to fresh solidified half-strength MS media, supplemented with 1% (w/v) sucrose. Unless mentioned otherwise, after another 7 days of growth, the seedlings of indicated genotypes were rotated by 90° and the gravitropic curvature was measured at different time points and scored in categories of 20°.

### Leaf venation analyses

Venation patterns were recorded from second and third leaves of 13-day-old seedlings of the indicated genotypes. Fixation and dehydration of plant tissue was done as described (Sieburth, 1999). Briefly, the plant tissue was immersed overnight in a 3:1 mixture of ethanol: acetic acid and then dehydrated through 80%, 90%, 95%, and 100% (v/v) ethanol. For clearing, the tissue was incubated in saturated chloral hydrate solution (2.5 g mL^−1^) overnight. The tissue was visualized and imaged by a Zeiss Axio zoomV16 microscope system. Leaf venation analyses were performed on second and third leaves of each genotype using LIMANI (Dhondt et al., 2012).

### Auxin transport

Polar auxin transport assays were performed as previously described (Ruegger et al., 1998). In short, 4-cm-long inflorescence stems of 6-week-old plants were excised and the apical end was placed into a 1.5-mL microfuge tube containing 50 µL of labeling solution supplemented with half-strength MS liquid media, supplemented with 1% (w/v) sucrose, and 0.06 nCi mL^−1^ of [^3^H]-IAA (15–30 Ci mmol^−1^; Biotrend; ART 0340) at pH 5.7. The excised stems were incubated in the solution for different time points. A 0.8-cm section from the basal end of the labeled stem was excised and placed in a vial containing 2 mL of scintillation cocktail and incubated overnight before radioactivity was measured by scintillation counting.

### Hormone measurements

Roots and shoots of 2-week-old seedlings grown in 8-h light/16-h dark condition were excised and collected separately. Auxin measurements were done as previously described (Eggert and von Wiren, 2017).

### Gene expression analyses

RNA extraction, cDNA synthesis, and RT–qPCR analyses were performed as previously described (Laha et al., 2015). Seedlings were grown vertically on sterile solidified [0.8% (w/v) phytagel], half-strength MS media, supplemented with 1% (w/v) sucrose for 11 days in 8-h light/16-h dark condition, then transferred to liquid half-strength MS media (pH 5.7), supplemented with 1% (w/v) sucrose and allowed to grow for another 2 days before harvesting. Total RNA extraction was performed using the RNeasy Plant Mini Kit (Qiagen). For cDNA synthesis, 1 µg of RNA was treated with DNase I. The reverse transcription was performed according to the manufacturer’s instructions (Roboklon; AMV Reverse Transcriptase Native). SYBR Green reaction mix (Bioline; Sensimix SYBR No-ROX kit) was used in a Bio-Rad CFX384 real-time system for RT–qPCR. The results were evaluated using the Bio-Rad CFX Manager 2.0 (admin) system. *PP2AA3* and β*-TUBULIN* were used as reference genes.

### In vitro kinase assay

Recombinant enzymes were purified as described for the yeast Sfh1 protein (Schaaf et al., 2006). InsP_6_ kinase assays were performed by incubating enzymes in 15-µL reaction volume containing 20-mM HEPES (pH 7.5), 5-mM MgCl_2_, 5-mM phosphocreatine, 0.33 units creatine kinase, 12.5-mM ATP, 1-mM InsP_6_, and 1-mM DTT. The reaction was incubated at 28°C for 4 h, separated by PAGE and stained by toluidine blue (Losito et al., 2009). The InsP_4a_ kinase assay with recombinant ITPK1 was performed using the same reaction conditions described above. Here, InsP_4a_ was purified from [^3^H]- *myo*-inositol labelled *itpk1* plants as described earlier (Blüher et al., 2017).

### In vitro radioligand-binding-based reconstitution assays

The in vitro binding assays were performed as previously described (Laha et al., 2016). InsP_5_ isomers were obtained from Sichem (Bremen, Germany), 5-InsP_7_ was synthesized as described previously (Pavlovic et al., 2016), and [^3^H]-InsP_6_ was purchased from Biotrend (ART-1915-10). His_8_-MBP-IAA7 was isolated using a protocol used for protein purification of the yeast Sfh1 (Schaaf et al., 2006). ASK1–TIR1 was purified from insect cells as described (Tan et al., 2007). Insect cell-purified ASK1–TIR1 was incubated with recombinant His_8_-MBP-IAA7 at a molar ratio of 1:3 and in presence of 0.1-μM IAA. [^3^H]-InsP_6_ was added to the binding buffer consisting of 50-mM Tris–HCl, pH 7.5, 100-mM NaCl, 10-mM imidazole, 10% (v/v) glycerol, 0.1% (v/v) Tween-20, and 5-mM 2-mercaptoethanol (freshly added) in a total volume of 0.5 mL. The reaction mixture was then incubated at 4°C for 2 h. Then 30 μL of Ni-NTA resin was added, the reaction was vortexed briefly and incubated further at 4°C for 3 h. A total of 10-mL ice-cold washing buffer (reaction buffer without 2-mercaptoethanol) was added to individual reactions and after 20 s Ni–NTA beads were filtered with 2.4-μm glass fiber filters (25 mm; Whatman Cat No 1822 025) using a filtration system (model FH225V, Hoefer, San Francisco), before analyzing filter membranes by scintillation counting. Data collection and evaluation of IC_50_ was carried out as described (Laha et al., 2016).

### Protein extraction and immunoblots from *A. thaliana*

For the detection of endogenous TIR1 levels, total proteins were extracted in 6M Urea from 2-week-old indicated Arabidopsis plants. The extract was centrifuged at 15,000*g* for 10 min at 4°C. The supernatant was mixed with Laemmli loading dye to final 1×. The proteins were separated on a 7.5% (w/v) sodium dodecyl sulfate–PAGE (SDS–PAGE), electroblotted onto a polyvinylidene difluoride (PVDF) membrane and immunoblot was performed with anti-TIR1 (Agrisera) or anti-actin (Abiocode) antibodies. Blots were developed with help of an ECL kit (Biorad) and images acquired using the Alpha imagequant system (GE).

For co-immunoprecipitation assays, 3-week-old plants of *ITPK1–G3GFP* (compl. line#7) or a transgenic line expressing GFP alone were used. Leaf tissues were homogenized (w/v) in RIPA buffer [5-mM Tris–HCl, pH 7.5, 150-mM NaCl, 10-mM MgCl_2_, 1-mM EDTA, 1% (w/v) NP-40, 1-mM sodium deoxycholate] containing 1x plant protease inhibitors (Sigma). The supernatant was clarified through a fine mesh (100 µm) and centrifuged at 5,000*g* for 10 min at 4°C. The supernatant was then pre-cleared for 1 h with 25 µL of IgG agarose beads (Sigma) at 4°C with constant rotation. The beads were collected by centrifugation and washed three times with RIPA buffer. To the supernatant, 25 µL of anti-GFP agarose beads (Biobharati) were added and kept at rotation overnight at 4°C. On the following day, the anti-GFP agarose beads were washed as described for the IgG-beads. The anti-GFP-agarose beads were then resuspended in 100 µL of 1× Laemmli loading dye and separated on 7.5% (w/v) SDS–PAGE gel. After transfer to a PVDF membrane and blocking, immunoblots were performed with anti-GFP (Biobharati) or anti-TIR1 antibodies (Agrisera). Images were acquired as described above.

### BiFC assays in *N. benthamiana*

For BiFC assays, the indicated agrobacterium strains were cultured overnight in LB nutrient broth containing appropriate antibiotics. The cultures were then pelleted and resuspended in equal volumes of induction medium (10-mM MgCl_2_, 10-mM MES, pH 5.6) also containing 100-μM acetosyringone (Sigma) and maintained at room temperature for 4 h. Indicated combinations of agrobacterium suspensions were mixed at similar cell densities and infiltrated into *N. benthamiana* leaves. About 48-h post-infiltration, tissue sections from the infiltrated leaves were viewed under a SP8 confocal microscope (Leica-microsystems). Images were acquired with both bright field and YFP filters (40× oil objective, Excitation 488 nm, collection bandwidth 495–544 nm; Laser intensity at 42% and gain set at 38 were used for all image acquisition).

### Elemental analysis

Whole shoot samples were dried for 48 h at 65°C and digested with nitric acid in polytetrafluoroethylene vials in a pressurized microwave digestion system (UltraCLAVE IV, MLS GmbH). Total phosphorus concentrations were analyzed by sector-field high-resolution inductively coupled plasma mass spectrometry (ELEMENT 2, Thermo Fisher Scientific, Germany). Element standards were prepared from certified reference standards from CPI-International (USA).

### Statistical analysis

One-way analysis of variance followed by a Dunnett’s or Tukey’s post hoc test on SPSS 24 (IBM), Chi-squared test and Student’s *t* test were applied for statistical analysis.

### Accession numbers

Sequence data from this article can be found in the GenBank/EMBL databases under the following accession numbers: *ITPK1* (At5g16760), *ITPK2* (At4g33770), *IPK1* (At5g42810), *PP2AA3* (At1g13320), *IAA19* (At3g15540), *IAA5* (At1g15580), *ARF19* (At1g19220), *IAA29* (At4g32280), *TIR1* (At3g62980), and *ß-TUBULIN* (At5g62700).

## Supplemental data

The following materials are available in the online version of this article.

**Supplemental Figure S1**. ITPK1 regulates seedling growth and development in Arabidopsis, and a C-terminal G3GFP fusion of ITPK1 does not compromise ITPK1 functions.

**Supplemental Figure S2.** InsP analyses of Arabidopsis *itpk1* T-DNA insertion lines.

**Supplemental Figure S3.** Thermomorphogenic responses and primary root growth are controlled by ITPK1.

**Supplemental Figure S4.** Auxin-related growth and developmental processes are not affected in Arabidopsis *pho2-1* plants.

**Supplemental Figure S5.** Role of ITPK1 in phosphorus accumulation and the effect of auxin.

**Supplemental Figure S6.** VIH2-deficient plants are not compromised in auxin perception.

**Supplemental Figure S7.** The *ipk1-1* mutant is defective in auxin perception and InsP/PP-InsP homeostasis.

**Supplemental Figure S8.** Binding of InsPs to the auxin-receptor complex.

**Supplemental Figure S9.** The *itpk2-2* lines are not defective in InsP synthesis and auxin responses.

**Supplemental Figure S10.** ITPK1 and ITPK2 act redundantly to control root gravitropism.

**Supplemental Figure S11.** Structural considerations of InsP binding of the auxin receptor complex and stability of TIR1 in *itpk1* plants.

**Supplemental Table S1.** Primer list.

**Supplemental Table S2.** MRM parameter settings for InsPs and PP-InsPs.

## Supplementary Material

kiac425_Supplementary_Data

## Notes

N.P.L., R.F.H.G., E.R., D.Q., N.J.P., R.S., Y.W.D., P.G., and D.L. performed the research and analyzed data. H.M. and N.Z. contributed important reagents. R.Y., Y.M.M., V.G., N.Z., N.v.W., A.S., S.B., H.J.J., and G.S analyzed the data. D.L. and G.S. designed the research. N.v.W., A.S., S.B., H.J.J., D.L., and G.S. supervised the research. D.L. and G.S. wrote the paper with input from all the authors.

The author responsible for distribution of materials integral to the findings presented in this article in accordance with the policy described in the Instructions for Authors (https://academic.oup.com/plphys/pages/general-instructions) is: Gabriel Schaaf (gabriel.schaaf@uni-bonn.de).
