# Correction to: INOSITOL (1,3,4) TRIPHOSPHATE 5/6 KINASE1-dependent inositol polyphosphates regulate auxin responses in Arabidopsis

**DOI:** 10.1093/plphys/kiae394

**Published:** 2024-08-06

This is a correction to: Nargis Parvin Laha, Ricardo F H Giehl, Esther Riemer, Danye Qiu, Naga Jyothi Pullagurla, Robin Schneider, Yashika Walia Dhir, Ranjana Yadav, Yeshambel Emewodih Mihiret, Philipp Gaugler, Verena Gaugler, Haibin Mao, Ning Zheng, Nicolaus von Wirén, Adolfo Saiardi, Saikat Bhattacharjee, Henning J Jessen, Debabrata Laha, Gabriel Schaaf, INOSITOL (1,3,4) TRIPHOSPHATE 5/6 KINASE1-dependent inositol polyphosphates regulate auxin responses in Arabidopsis, Plant Physiology, Volume 190, Issue 4, December 2022, Pages 2722–2738, https://doi.org/10.1093/plphys/kiac425

The authors identified a mistake in calculating the absolute concentrations of inositol(pyro)phosphates derived from CE-ESI-MS measurements reported in the originally published version of the manuscript. Due to a calculation error when determining concentrations per fresh biomass, the absolute values shown in the manuscript are approximately 30-fold lower than they should be. This mistake affects only panel C in Supplemental Figure S7. Since all conclusions made with respect to this figure referred to relative differences between genotypes, they remain unaffected by this mistake.

The corrected version of the figure is given below:

**Figure:**
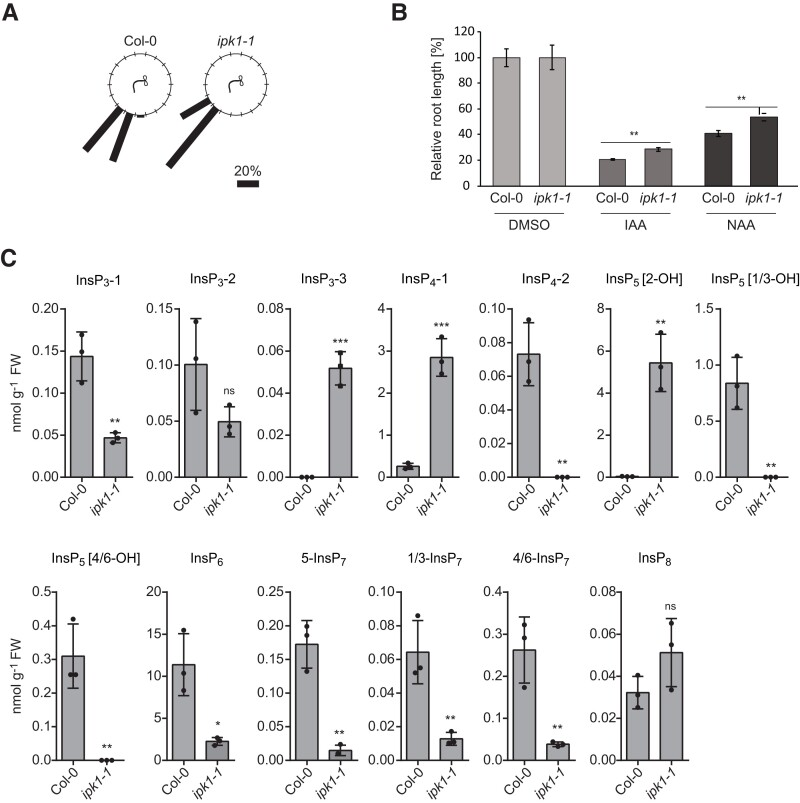


These details have been corrected only in this correction notice to preserve the published version of record. The authors sincerely apologize for this mistake and any confusion it may have caused.

