# Supplementary material for: INOSITOL (1,3,4) TRIPHOSPHATE 5/6 KINASE1-dependent inositol polyphosphates regulate auxin responses in Arabidopsis
Source: Plant Physiol. 2022 Sep 19;190(4):2722–38. doi: 10.1093/plphys/kiac425 (PMC9706486; doi:10.1093/plphys/kiac425)
Supplement: kiac425_Supplementary_Data [file kiac425_supplementary_data.pdf]

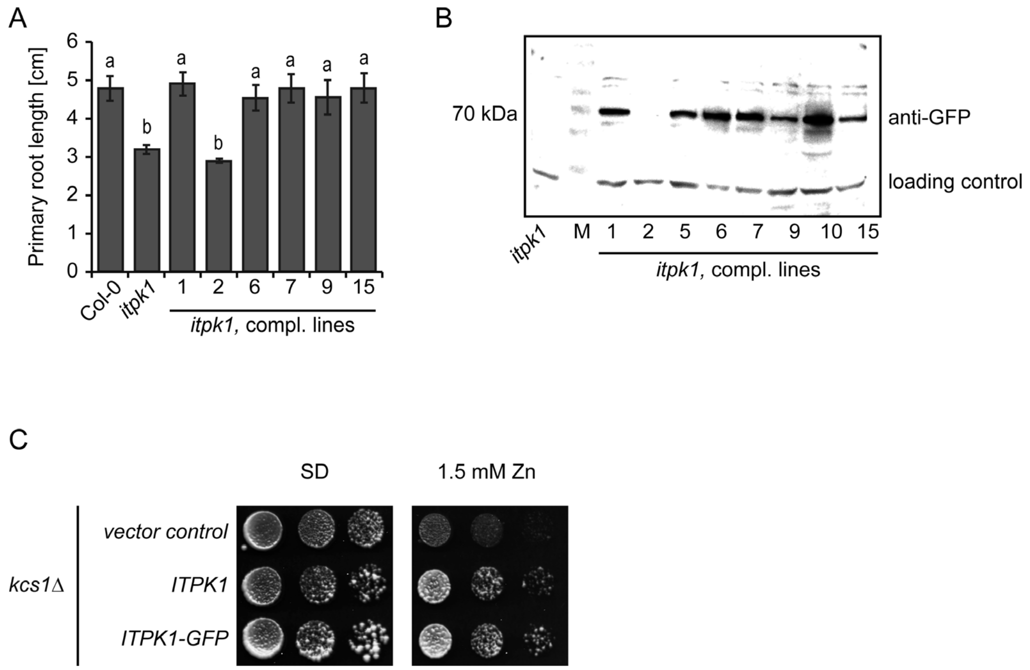

Supplemental Figure S1. ITPK1 regulates seedling growth and development in *Arabidopsis* and a C-terminal G3GFP fusion of ITPK1 does not compromise ITPK1 functions.

(A) Evaluation of primary root growth in wild-type (Col-0), *itpk1* mutant and independent *itpk1* lines complemented with a genomic ITPK1 fragment C-terminally fused to G3GFP. Seeds of indicated genotypes were surface sterilized and sown on sterile solidified half-strength MS media supplemented with 1% sucrose. Germinated seedlings were allowed to grow for 13 days and digitally recorded afterwards. Root lengths were evaluated by ImageJ. Error bars represent standard error of means (SEM.), $n \geq 7$. Letters indicate significance in one-way ANOVA) followed by Tukey's test (a and b, $P < 0.005$). The experiment was performed twice with similar results.
(B) Screening for *itpk1* complemented lines. Immunoblot analyses of soluble protein extracts from 2-week-old seedlings of the indicated genotype grown on sterile solidified half-strength MS media supplemented with 1% sucrose. An unspecific band was selected as a loading control.
(C) A C-terminal G3GFP fusion of ITPK1 does not compromise ITPK1 functions. ITPK1 or ITPK1-G3GFP were expressed from episomal plasmid pDR195 in a *kcs1Δ* yeast strain. Transformants were spotted either on selective minimal medium with appropriate supplements (SD, left), or SD medium and appropriate supplements containing 1.5 mM $ZnSO_4$ (right).

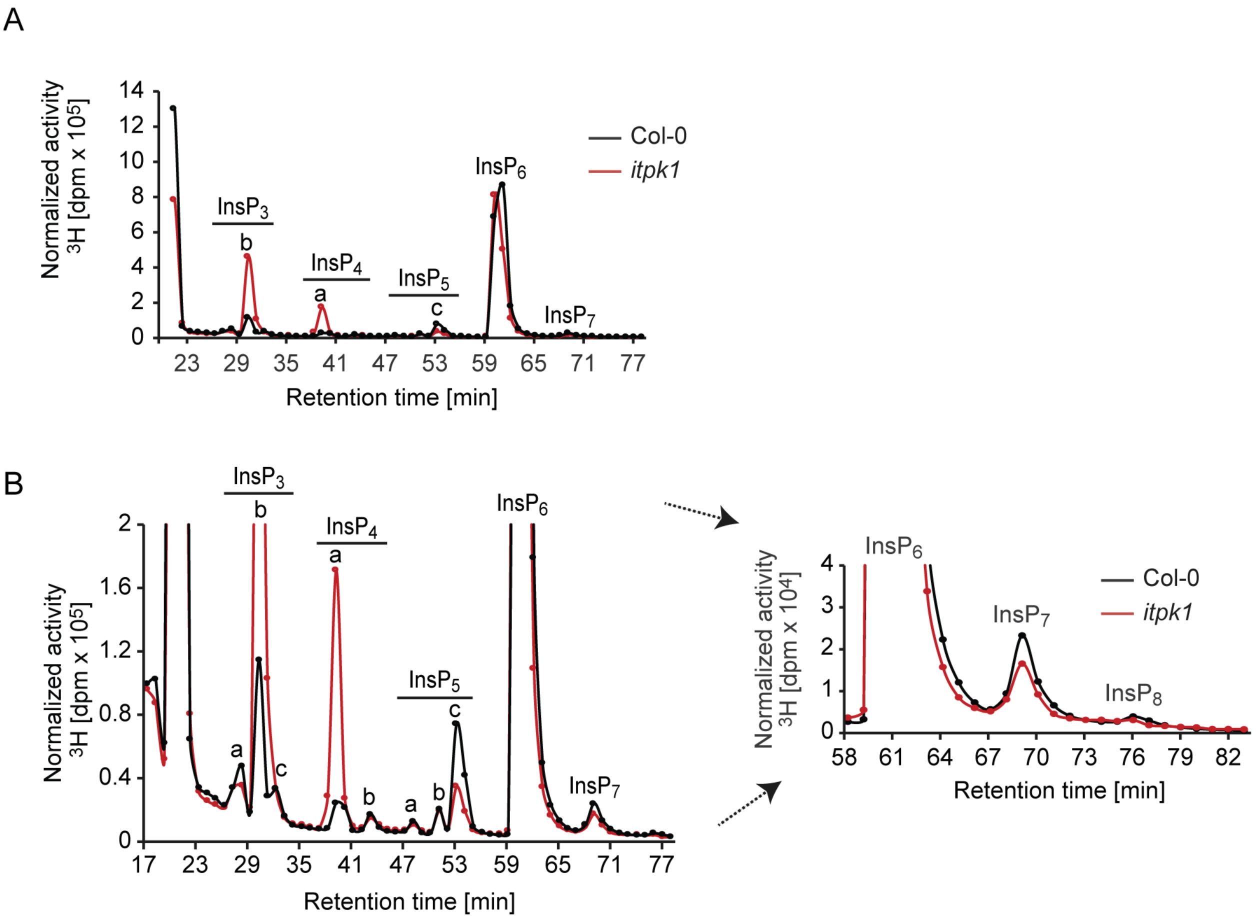

Supplemental Figure S2. InsP analyses of Arabidopsis *itpk1* T-DNA insertion lines.

(A) SAX-HPLC profiles of extracts of 3-week-old [^{3}H] inositol-labeled wild-type (Col-0, solid black line) and *itpk1* mutant (solid red line) seedlings. Activities obtained by scintillation counting of fractions containing the $InsP_2$-$InsP_8$ peaks are shown. InsP denotes inositol phosphate.

(B) Enlargement of the SAX-HPLC profiles of (A). The $InsP_6$-$InsP_8$ region is presented with arrows. The isomeric nature of $InsP_{3[a-c]}$, $InsP_{4\,[a,b]}$ is not yet solved. Based on published chromatographs (Stevenson-Paulik et al., 2005; Laha et al., 2015), $InsP_{5a}$ corresponds to $InsP_5$ [2-OH], $InsP_{5b}$ represents $InsP_5$ [4/6-OH] and $InsP_{5c}$ corresponds to $InsP_5$ [1-OH] or its enantiomer $InsP_5$ [3-OH]. InsP denotes inositol phosphate.

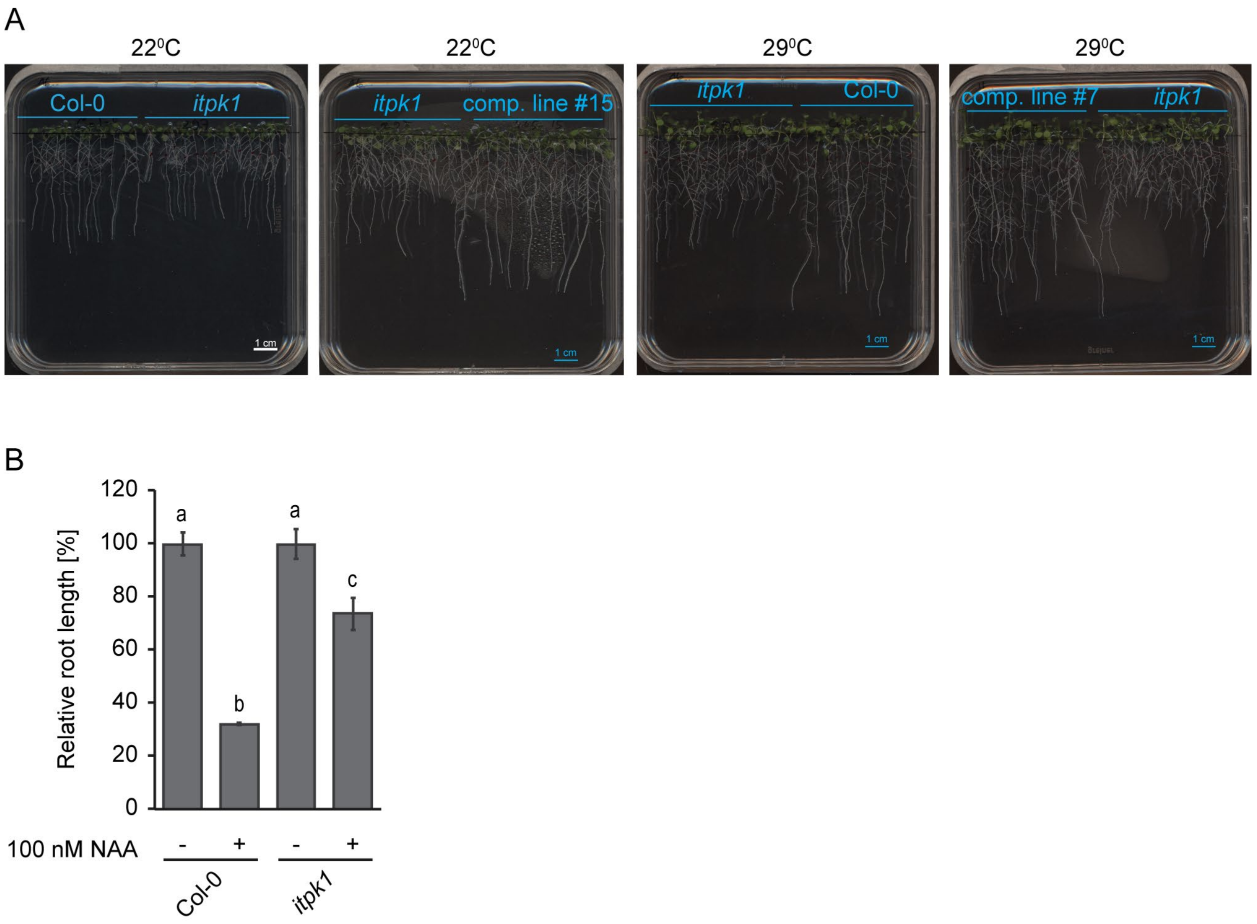

Supplemental Figure S3. Thermomorphogenic responses and primary root growth are controlled by ITPK1.
(A) Represenatitive plate pictures of designated genotypes grown on solidified half-strength MS media supplemented with 1% sucrose under control condition or at higher temperature. 5-day-old seedlings were kept at 22°C or shifted to 29°C and kept for 8 daysbefore picture was taken.
(B) Relative root length of wild-type (Col-0) and *itpk1* mutant treated with 100 nM 1-naphthaleneacetic acid (NAA). Seeds of indicated genotypes were surface sterilized and sown on sterile solidified half-strength MS media supplemented with 1% sucrose with or without NAA. Germinated seedlings were allowed to grow for 16 days. Root lengths were evaluated by ImageJ. Letters depict significance in a one-way ANOVA followed by Tukey's test (a and b, $P < 0.001$; b to c, $P < 0.001$; a to c, $P < 0.01$). Data are shown as means ± SEM, n=10-29.

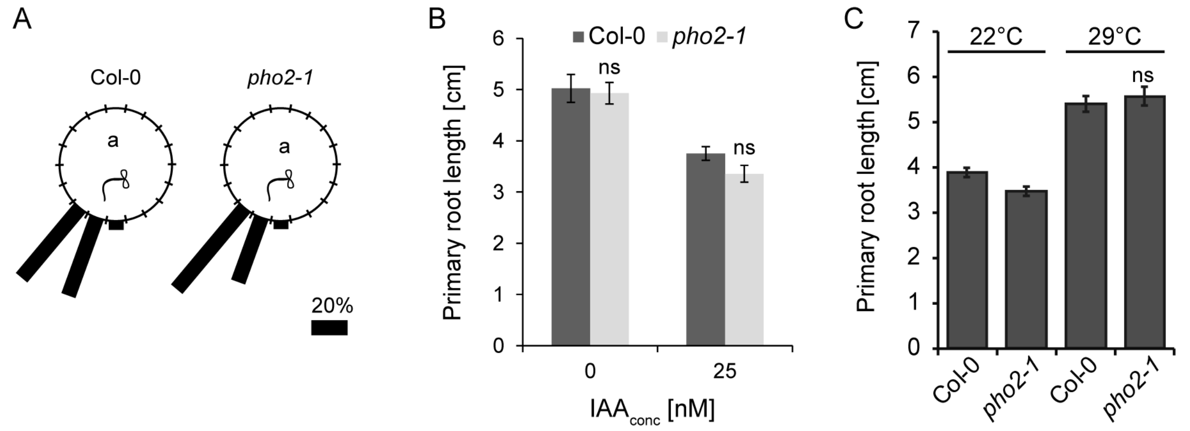

Supplemental Figure S4. Auxin-related growth and developmental processes are not affected in Arabidopsis *pho2-1* plants.

(A) Root gravitropism of seedlings of wild-type (Col-0) and *pho2-1* mutant after 90° reorientation. 7-day-old seedlings of Col-0 and *pho2-1* were transferred to solidified half-strength MS media supplemented with 1% sucrose and after another 12 days of growth, the seedlings were rotated by 90° and the gravitropic curvature was measured after 16 h. The distribution of data was analyzed using a $\chi 2$ test (number of seedlings $n \geq 22$, groups contained at least 4% of total seedlings per genotype). Same letter (a) denotes that no significant differences at $P < 0.05$ were detected.
(B) Effect of auxin on the primary root length of the phosphorus overaccumulator mutant *pho2-1*. 6-day-old seedlings of wild-type (Col-0) and *pho2-1* were transferred to solidified half-strength MS media supplemented with 1% sucrose and with 0 or 25 nM indole-3-acetic acid (IAA) and 625 µM phosphate. Primary root length was measured 7 days after transferring plants to treatments. Bars show means ± SEM (n = 11) . No significant differences at $P < 0.05$ were detected by two-tailed Student's *t*-test.
(C) 5-day-old seedlings of wild-type (Col-0) and *pho2-1* grown at 22°C grown on solidified half-strength MS media supplemented with 1% sucrose were kept at 22°C or shifted to 29°C. Root length was evaluated after 8 days by ImageJ. Error bars represent SEM, $n \geq 23$. No significant differences at $P < 0.05$ were detected by two-tailed Student's *t*-test.

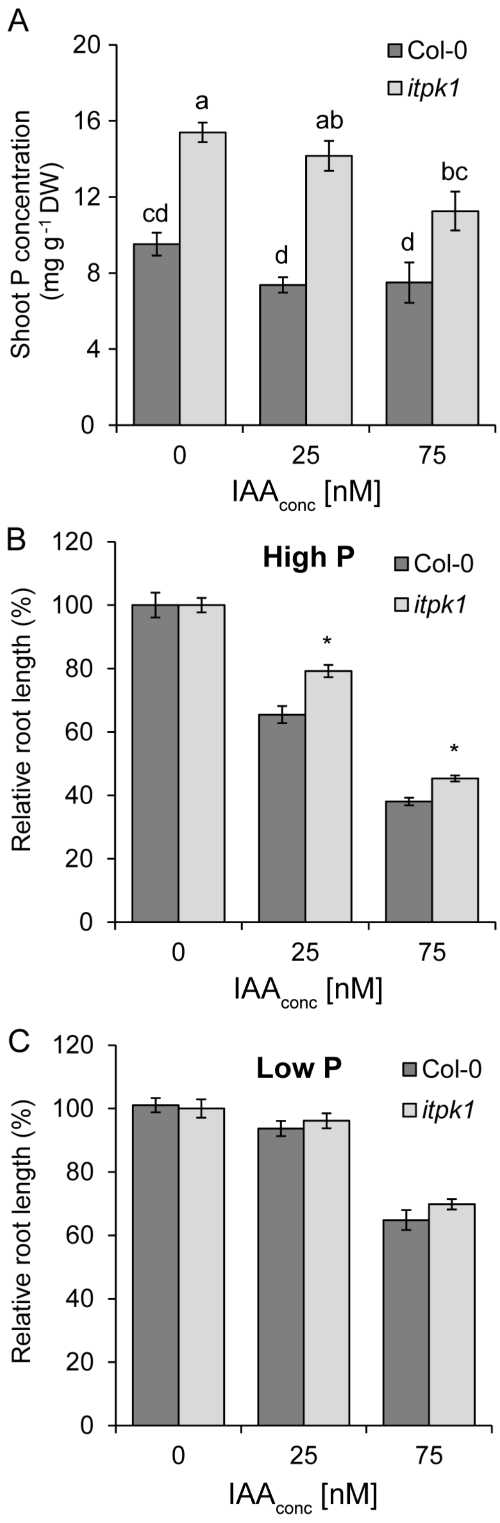

Supplemental Figure S5. Role of ITPK1 in phosphorus accumulation and the effect of auxin.

(A-C) Shoot phosphorous (P) concentration and relative primary root lengths of wild-type (Col-0) and *itpk1*mutant grown under increasing indole-3-acetic acid (IAA) concentrations. DW

denotes dry weight. 6-day-old seedlings were transferred to solidified half-strength MS media with 1% sucrose supplemented with 0, 25 and 75 nM IAA under 625 µM phosphate (High P) (*A* and *B*) or 10 µM phosphate (Low P) (C). Shoot phosphorus concentration (A) and relative primary root length (B and C) were assessed 7 days after transferring plants to treatments. Bars show means ± SEM (n = 4 replicates with 4 plants each for shoot phosphorus analysis and 15 individual plants for primary root length). Absolute values for phosphorus concentrations were compared by ANOVA and post-hoc Tukey test and different letters indicate significant differences at $P < 0.05$. Relative root growth was compared by pairwise Student's *t*-test and significant differences at $P < 0.01$ are indicated by single asterisk.

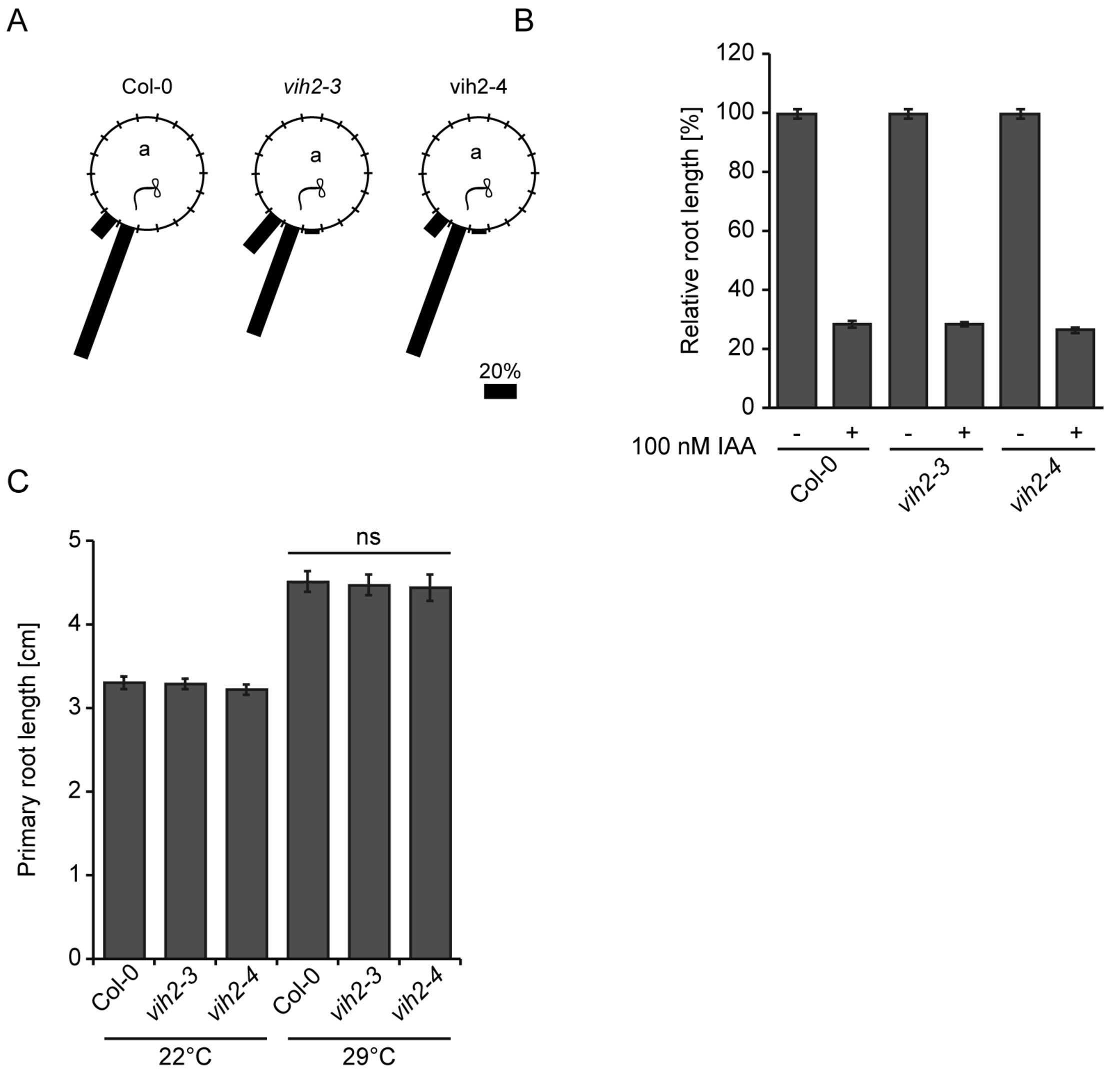

Supplemental Figure S6. VIH2-deficient plants are not compromised in auxin perception.

(A) Root gravitropism of seedlings of wild-type (Col-0), *vih2-3* and *vih2-4* mutants after 90° reorientation. 7-day-old seedlings of indicated genotypes grown on solidified half-strength MS media supplemented with 1% sucrose, were transferred to new solid media and after another 12 days of growth, the seedlings were rotated by 90° and the gravitropic curvature was measured after 16 h. The distribution of data was analyzed using a $\chi 2$ test (number of seedlings $n \geq 35$). Same letter denotes that no significant differences at $P < 0.05$ were detected. The experiment was repeated independently with similar results.
(B) Relative root length of wild-type (Col-0), *vih2-3* and *vih2-4* mutants treated with 100 nM indole-3-acetic acid (IAA). Seeds were surface sterilized and sown on sterile solidified half-strength MS media supplemented with 1% sucrose. 6-day-old seedlings were transferred to new media containing either 100 nM IAA or DMSO as control and scanned after 7 days. Root lengths were evaluated by ImageJ. Data are means ± SEM, n≥34. No significant differences at $P < 0.05$

were detected by two-tailed Student's *t*-test. The experiment was repeated independently with similar results.
(C) Primary root length analysis of seedlings of wild-type (Col-0), *vih2-3* and *vih2-4* mutants grown at higher temperatures. 5-day-old seedlings of designated genotypes were grown on solidified half-strength MS media supplemented with 1% sucrose at 22°C, then kept at 22°C or shifted to 29°C. Root length was evaluated after 8 days by ImageJ. Error bars represent SEM, $n \geq 25$. No significant differences at $P < 0.05$ were detected by two-tailed Student's *t*-test. The experiment was repeated independently with similar results.

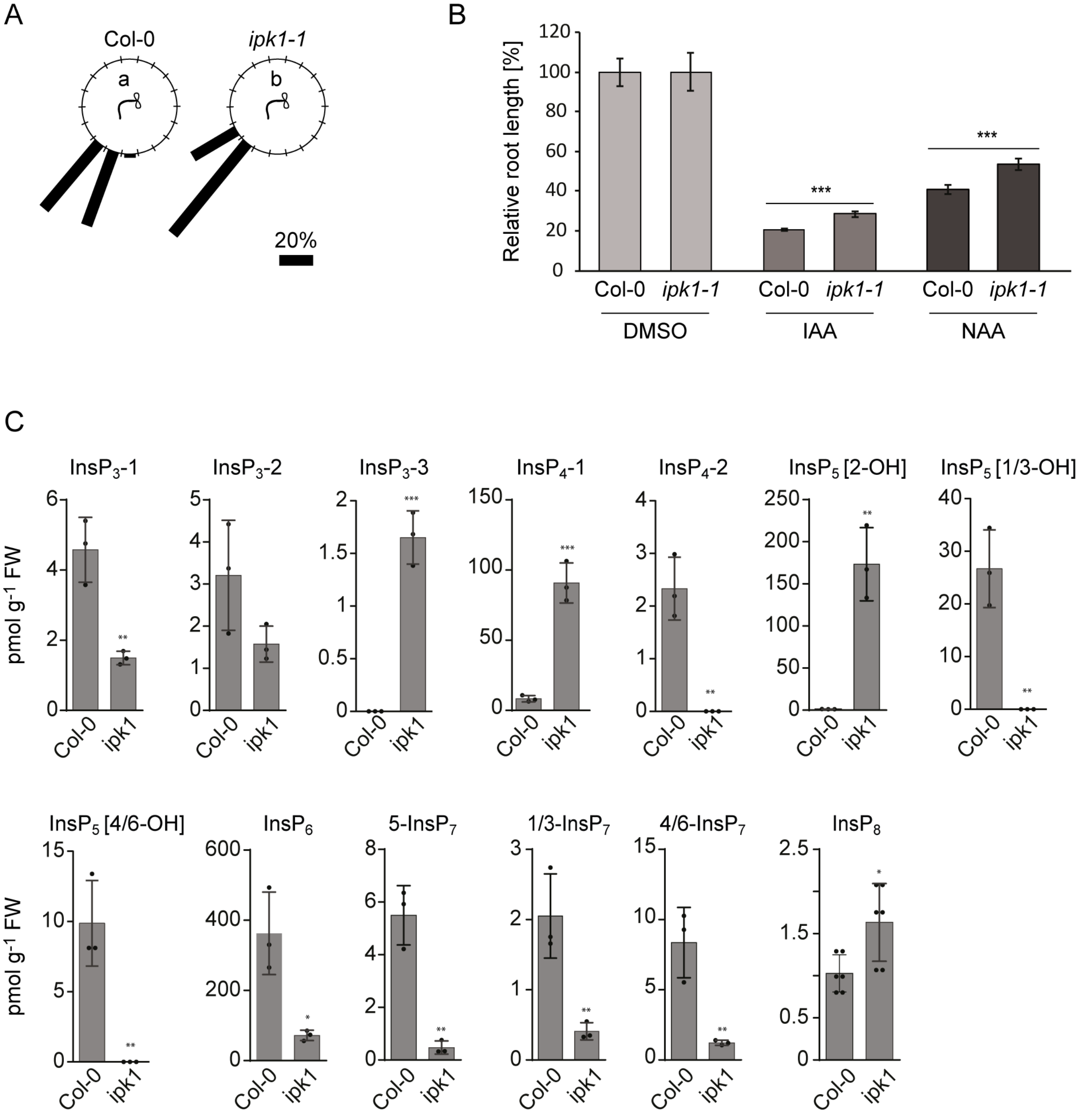

Supplemental Figure S7. The *ipk1-1* mutant is defective in auxin perception and InsP/PP-InsP homeostasis.

(A) Root gravitropism of seedlings of wild-type (Col-0) and *ipk1-1* mutant after 90° reorientation. 12-day-old seedlings of indicated genotypes grown on solidified half-strength MS media supplemented with 1% sucrose were rotated by 90° and the gravitropic curvature was measured after 16 h. The percentage of the seedlings in each category is represented by the length of the bar. FW denotes fresh weight. The distribution of data was analyzed using a $\chi 2$ test (number of seedlings $n \geq 20$). Means with different letters are significantly different, $P < 0.005$. The experiment was done independently with similar results.

(B) Relative root length of wild-type (Col-0) and *ipk1-1* mutant treated with IAA and NAA . Seeds of Col-0 and *ipk1-1* (Laha et al., 2015) were surface sterilized and sown on sterile solidified half-strength MS media supplemented with 1% sucrose with or without auxin, indole-3-acetic acid (IAA) or 1-naphthaleneacetic acid (NAA). Germinated seedlings were allowed to grow for 9 days. Root lengths were evaluated by ImageJ. Error bars present SEM, n≥10. Asterisks indicate statistical differences between wild-type and *ipk1-1* plants when treated exogenously with either IAA or NAA (two-tailed Student's *t*-test; ***$P < 0.001$).

(C) CE-ESI-MS analysis of inositol polyphosphate levels of shoots of 35-day-old Arabidopsis wild-type (Col-0) and the *ipk1* mutant. Plants were cultivated in hydroponics with sufficient supply of all nutrients. Data are means ± SEM (n = 3 biological replicates). *P < 0.05, **P < 0.01 and ***P < 0.001 , according to two-tailed Student's *t*-test (*ipk1* vs Col-0). The same Col-0 extracts used in this analysis also served as control in a previous study (Riemer et al., 2021). Col-0 and *ipk1* plants were grown together in the same experiment and samples harvested, extracted and analyzed at the same time. InsP denotes inositol phosphate.

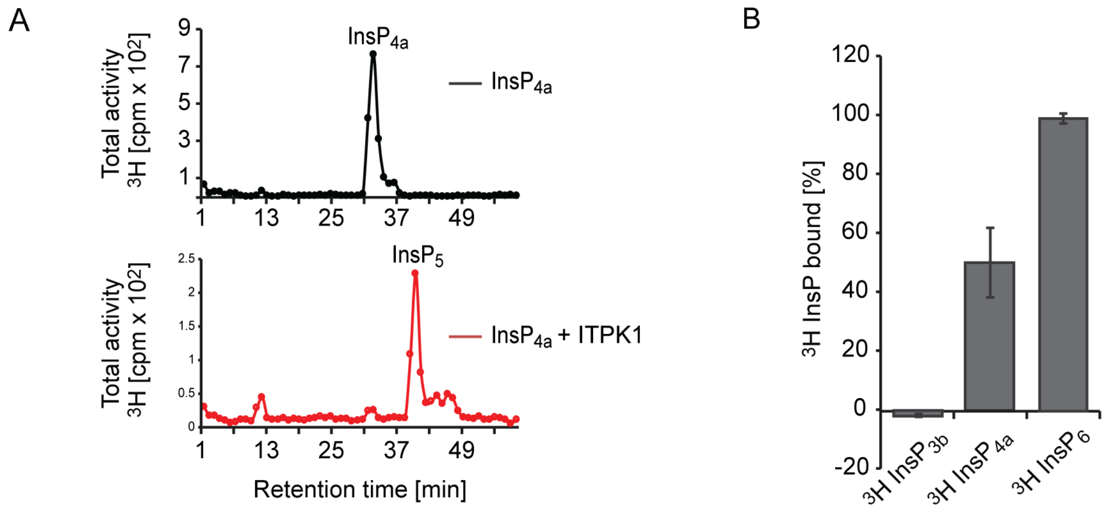

Supplemental Figure S8. Binding of InsPs to the auxin-receptor complex.

(A) SAX-HPLC of ITPK1 kinase reaction. [^{3}H]-InsP4a was purified from [^{3}H] inositol-labeled *itpk1-2* plants and incubated with recombinant ITPK1 and ATP. The kinase product was resolved by SAX-HPLC. InsP denotes inositol phosphate.
(B) Direct binding of [^{3}H]-$InsP_{3b}$, [^{3}H]-$InsP_{4a}$, and [^{3}H]-$InsP_6$ to the TIR1/ASK1/IAA7 auxin receptor complex. A total activity of 2000 cpm was used for each [^{3}H]-labeled InsP species. [^{3}H]-$InsP_{3b}$ and [^{3}H]-$InsP_{4a}$ were purified and desalted from [^{3}H]-*myo*-inositol labeled seedlings of the *itpk1-2* mutant and [^{3}H]-$InsP_6$ from Col-0 seedlings. Values show means ± SEM (n = 2). InsP denotes inositol phosphate.

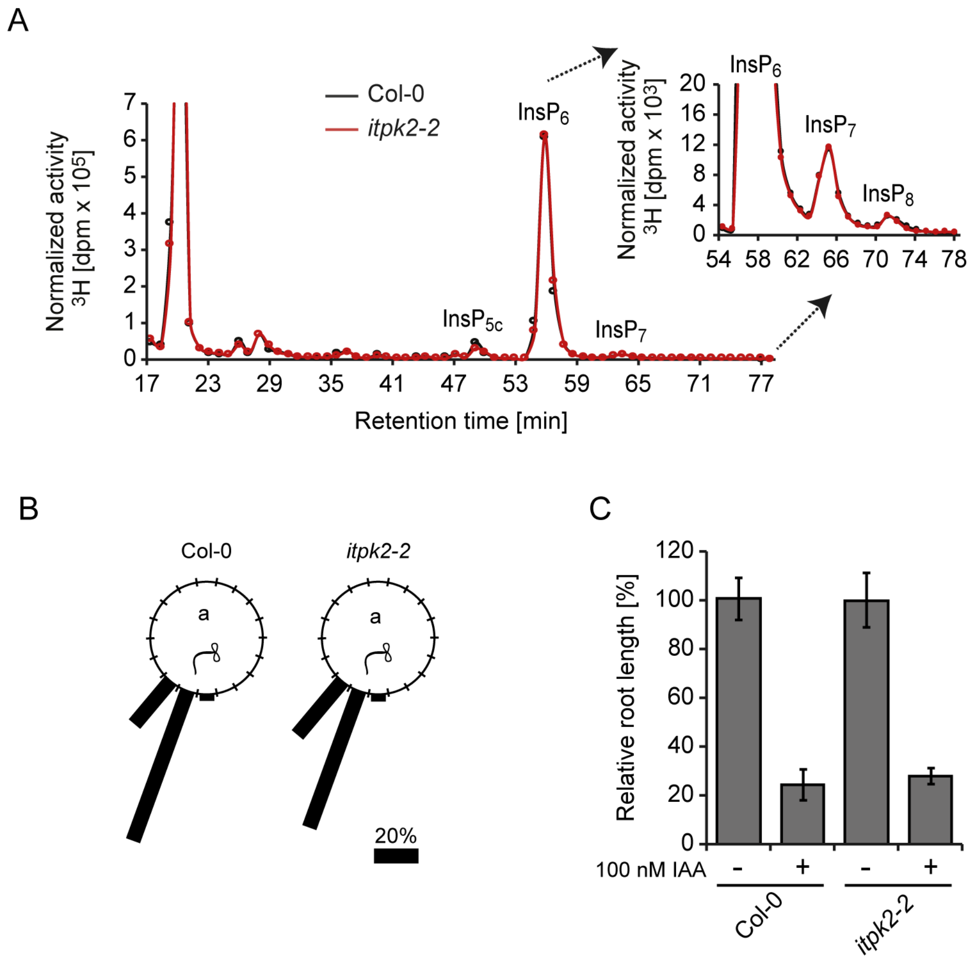

Supplemental Figure S9. The *itpk2-2* lines are not defective in InsP synthesis and auxin responses.

(A) The *itpk2-2* line appears not to be compromised in InsP synthesis. Extracts of designated [^{3}H] inositol-labeled Arabidopsis seedlings were resolved by SAX-HPLC. Activities obtained by scintillation counting of fractions containing the $InsP_2$-$InsP_8$ peaks are presented.

(B) Root gravitropism of seedlings of wild-type (Col-0) and *itpk2-2* plants after 90° reorientation. 7-day-old seedlings grown on solidified half-strength MS media supplemented with 1% sucrose were transferred to new media and after another 12 days of growth, the seedlings of Col-0 and *itpk2-2* were rotated by 90° and the gravitropic curvature was measured after 16 h. The distribution of data was analyzed using a $\chi 2$ test (number of seedlings $n \geq 35$). No significant differences at $P < 0.05$ were detected.

(C) Relative root length of wild-type (Col-0) and *itpk2-2* mutant treated with 100 nM indole-3-acetic acid (IAA). Seeds of indicated genotypes were surface sterilized and sown on sterile solidified half-strength MS media supplemented with 1% sucrose. 6-day-old seedlings were transferred to new plates containing either 100 nM IAA or DMSO as control and scanned after 7 days. Root lengths were evaluated by ImageJ. Data are means ± SEM, n=37. The experiment was repeated independently with similar results. No significant differences at $P < 0.05$ were detected by two-tailed Student's *t*-test.

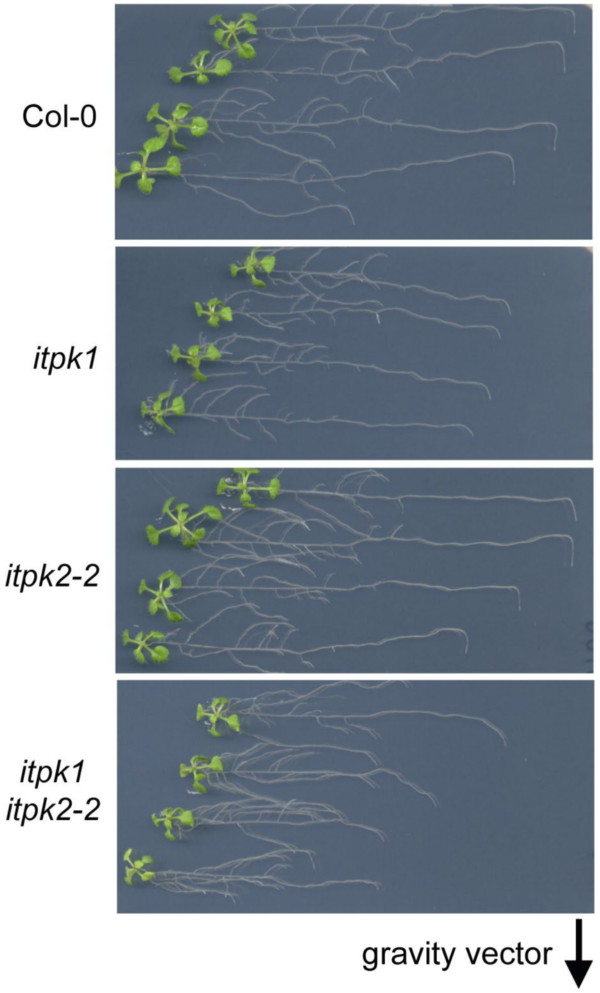

Supplemental Figure S10. ITPK1 and ITPK2 act redundantly to control root gravitropism.

Root gravitropism of wild-type (Col-0), *itpk1*, *itpk2* and *itpk1itpk2* double knockout plants. 7-day-old seedlings grown on solidified half-strength MS media supplemented with 1% sucrose were transferred to new media and after another 7 days of growth, the seedlings of indicated genotypes were rotated by 90° and the gravitropic curvature was measured after 28 h.

A

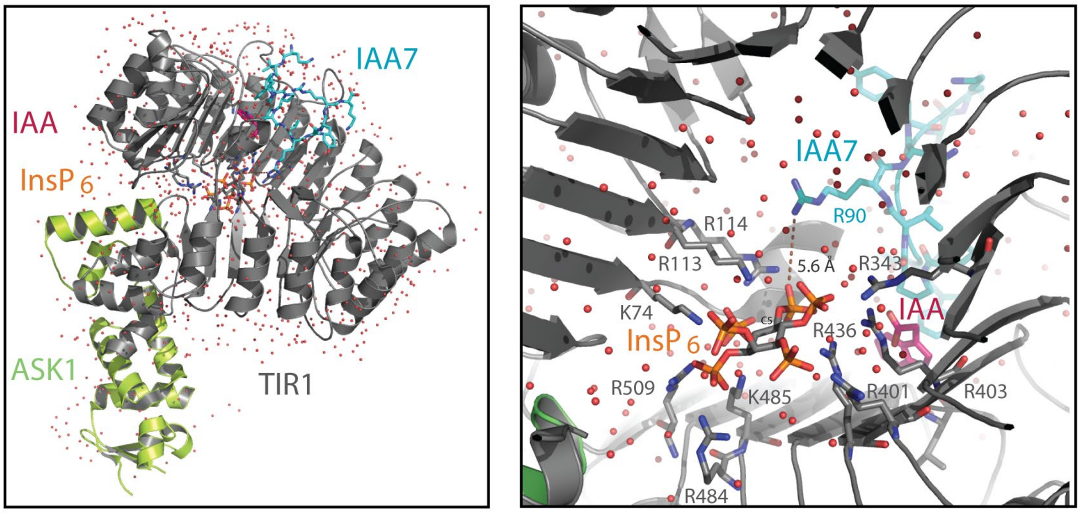

B

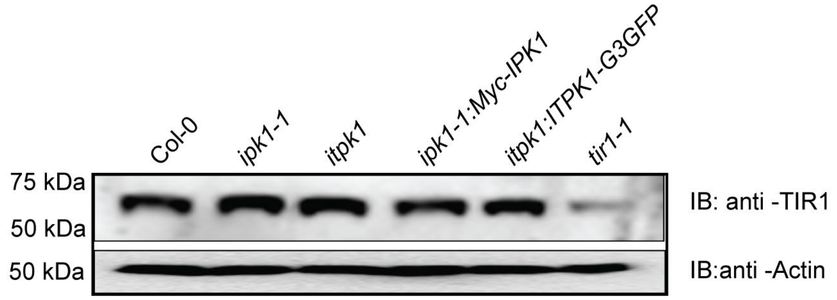

Supplemental Figure S11. Structural Considerations of InsP Binding of the Auxin Receptor Complex and Stability of TIR1 in *itpk1* Plants

(A) Structural considerations of $InsP_6$ binding by the auxin-receptor complex.

Richardson diagram of the TIR1-ASK1-IAA7 degron complex bound to indole-3-acetic acid (IAA) and $InsP_6$ (PDB ID: 2P1Q). TIR1 (gray), ASK1 (lime green), the IAA7 degron (cyan stick), $InsP_6$ (orange stick), and IAA (magenta stick) are presented. TIR1 residues engaging in polar contacts with $InsP_6$ are depicted as sticks. Note their anisotropic distribution distal and proximal to the hormone binding pocket. The distance between IAA7 degron residue Arg 90 and the closest phosphate of $InsP_6$ (i.e. at position C5) is indicated and suggests a strong interaction with the inositol pyrophosphate moiety of 5-$InsP_7$ - provided that both $InsP_6$ and $InsP_7$ occupy the InsP binding pocket of the auxin receptor complex in a similar fashion. Images were generated with PyMOL (The PyMOL Molecular Graphics System, Version 0.99 Schrödinger, LLC).

(B) Levels of the auxin receptor component TIR1 remain unaffected in *ipk1-1* and *itpk1* plants. Immunoblot analysis of TIR1 in whole plant extracts of 14-day-old seedlings of wild-type (Col-0), *ipk1-1*, *itpk1-2*, the respective complemented lines *ipk1-1: Myc-IPK1*, *itpk1-2: ITPK1-G3GFP*, and the *tir1-1* mutant. The bottom panel shows immunoblot of same extracts with anti-actin antibodies as a loading control. Migration position of molecular weight standards (in kDa) is shown at the left of each panel.

Supplemental Tables

Supplemental Table S1. Primer list.

Primer list for PCR-based characterization of T-DNA insertion lines.

Mutant lines	Sense Primer (5'-3')	Anti-sense Primer (5'-3')
itpk1	GCTTCCTATTATATCTTCCCAAATTACCA ATACA (LB2_SAIL)	CATGGCTTTGCAAAACTCG GAAG
itpk2-2	GCTTCCTATTATATCTTCCCAAATTACCA ATACA (LB2_SAIL)	TCGGTTATGTTTAAACGCC AAC

Primers used for RT-qPCR-analyses.

Gene	Sense Primer (5'-3')	Anti-Sense Primer (5'-3')
IAA29	GGGTGCTGCGTCTTGTTTGGGT	TCCTCGTTGGGCTGGCCATT
IAA5	GTCGTCTCCGGTGAGTCCATCT	AAACCGGTGGCCAACCCACAA
PP2AA3	GGCAGAAGTTCGGATAGCAG	CAATGCAGATCTGACGTGCT
IAA19	TGGCATCGGTGTGGCCTTGAA	ACATCACCGGCGAGCATCCAGT
ARF19	TTGAGCGCGCAAGCAATCCG	TGCCTTGACTGATCCGGCTCTCA
ITPK1	AGTTGGATACGCACTCGCAGCC	TGCCTCGTTGCCTTGAGTGTTCG
ITPK2	AACGCAGACTTGGACCCTCGTG	AAGCGCCTCGAGGAAAGGCT
GH3.2	AACGAAGCCATCCTCTGCTCCGA	TGCGCCGAGTCGGAGAACTT
LBD33	TGCAACCGCGACTGCGTCTT	TGTGAACCGCTGCGAAATGGGA

Primer list to clone into the pENTR-D-TOPO vector.

Gene	Sense Primer (5'-3')	Anti-Sense Primer (5'-3')
ITPK1	caccATGTCAGATTCAATCCAGGAAAG	TCAGACATGATTCTTCTTAGTG
ITPK2	caccATGTTTGGGACTCTTGCTTCCGGC	TCATTTACAATGTTGTCTCTT
ITPK3	caccATGTACTGGCAGCAAATTGGA	TTAGTATTGATCTGCCAAAGCT
ITPK4	caccATGAAAGGGGTTCTACTTGACGA	TCAATGCTTCTCTTGGACATGTT
IPK2a	caccATGCAGCTCAAAGTCCCTGAAC	CTAAGAATCTGCAGACTCATC
IPK2b	caccATGCTCAAGGTCCCTGAACA	CTAGCGCCCGTTCTCAAGTAGG
IPK1	caccATGGAGATGATTTTGGAGGAGA	ttgcggccgcTTAGCTGTGGGAAGGTTTTGA G

Primers employed for site-directed mutagenesis.

Gene	Mutation	Primer sequence (5'-3', only sense orientation is listed)
ITPK1	K188A	CATGGTGGTGTGATCTTTgcGGTCTATGTGGTTGGAGATC
ITPK1	D288A	GCTAATAGGTACCTTATAATTGcTATTAACTACTTTCCTGG
ITPK2	K260A	AATCATGGTGGAGTTATGTTCgcGGTATTTGTGGTGGGTGATGTTA
ITPK2	D355A	GCAAAAACGTGTTTTATGTTATTGcCATCAACTATTTTCCTGGT

Specific mutations are in lower cased. Corresponding mutations in the final plasmids were confirmed by sequencing.

Primers employed to clone into the pET28- His_8-MBP bacterial expression vector

Gene	Primer Sequence (5'-3')
ITPK1	AAggatccATGTCAGATTCAATCCAGGAAAG (sense)<br>TgtcgacTCAGACATGATTCTTCTTAGTG (antisense)
ITPK2	AAgaattcATGTTTGGGACTCTTGCTTCCGG (sense)<br>TgtcgacTCATTTACAATGTTGTCTCTTCT (antisense)
hITPK1	AggatccATGCAGACCTTTCTGAAAGGGAA (sense)<br>tGCGGCCGCCTACTGGGAGGAGGCCTTGGTGG (antisense)
IAA7	AggatccATGATCGGCCAACTTATGAACCT (sense)

	TgcggccgcTCAAGATCTGTTCTTGCAGTAC (antisense)
VIH2	AAggatccATGGAGATGGAAGAAGGAGCA (sense)<br>TTgcggccgcTTAGCTCCTTCCATTAGAAGAAG (antisense)

Primers for amplifying ITPK1 and IPK1 cDNAs

cDNA	AttB1 For	Attb1 Rev
ITPK1	5'-GGGGACAAGTTTGTACAAAAAAGCAGGCTCAATGTCAGATTCAATTC-3'	5'-GGGGACCACTTTGTACAAGAAAGCTGGGTATCAGACATGATTCTTCTT-3'
TIR1	5'-GGGGACAAGTTTGTACA AAAAAGCAGGCTCAATGCAGAAGCGAATAGCCTTGTCG-3'	5'-GGGGACCACTTTGTACA AGAAAGCTGGGTATTATAATCCGTTAGTAGTAATGAT

Supplemental Table S2. MRM parameter settings for InsPs and PP-InsPs

**Compound Name**	**Precursor Ion**	**Product Ion**	**dwell**	**Frag (V)**	**CE (V)**	**Cell Acc (V)**	**Polarity**
$[^{13}C_6]InsP_8$	411.9	362.9	80	166	10	1	Negative
$InsP_8$	408.9	359.9	80	166	10	1	Negative
$[^{13}C_6]InsP_7$	371.9	322.9	80	166	10	3	Negtaive
$InsP_7$	368.9	319.9	80	166	10	3	Negative
$[^{13}C_6]InsP_6$	331.9	487	80	166	10	1	Negative
$[^{13}C_6]InsP_6$	331.9	78.9	80	166	46	3	Negative
$InsP_6$	328.9	481	80	166	10	1	Negative
$InsP_6$	328.9	78.9	80	166	46	3	Negative
$[^{13}C_6]InsP_5$	292	504.7	80	166	10	1	Negative
$[^{13}C_6]InsP_5$	288.9	498.7	80	166	10	1	Negative
$InsP_4$	249	418.6	80	166	10	1	Negative
$InsP_3$	419	320.6	80	166	18	4	Negative

Supplemental References

Laha D, Johnen P, Azevedo C, Dynowski M, Weiss M, Capolicchio S, Mao HB, Iven T, Steenbergen M, Freyer M, Gaugler P, de Campos MKF, Zheng N, Feussner I, Jessen HJ, Van Wees SCM, Saiardi A, Schaaf G (2015) VIH2 Regulates the Synthesis of Inositol Pyrophosphate InsP(8) and Jasmonate-Dependent Defenses in Arabidopsis. Plant Cell **27:** 1082-1097

Riemer E, Qiu D, Laha D, Harmel RK, Gaugler P, Gaugler V, Frei M, Hajirezaei MR, Laha NP, Krusenbaum L, Schneider R, Saiardi A, Fiedler D, Jessen HJ, Schaaf G, Giehl RFH (2021) ITPK1 is an InsP6/ADP phosphotransferase that controls phosphate signaling in Arabidopsis. Mol Plant

Stevenson-Paulik J, Bastidas RJ, Chiou ST, Frye RA, York JD (2005) Generation of phytate-free seeds in Arabidopsis through disruption of inositol polyphosphate kinases. Proc Natl Acad Sci U S A **102:** 12612-12617
